# General‐Purpose Ultrasound Neuromodulation System for Chronic, Closed‐Loop Preclinical Studies in Freely Behaving Rodents

**DOI:** 10.1002/advs.202202345

**Published:** 2022-10-19

**Authors:** Yehhyun Jo, Sang‐Mok Lee, Taesub Jung, Gijae Park, Chanhee Lee, Geun Ho Im, Seongju Lee, Jin Soo Park, Chaerin Oh, Geon Kook, Hyunggug Kim, Seongyeon Kim, Byung Chul Lee, Greg S.B. Suh, Seong‐Gi Kim, Jeongyeon Kim, Hyunjoo J. Lee

**Affiliations:** ^1^ School of Electrical Engineering Korea Advanced Institute of Science and Technology (KAIST) Daejeon 34141 Republic of Korea; ^2^ Korea Brain Research Institute (KBRI) Daegu 41068 Republic of Korea; ^3^ Department of Electrical Engineering Korea University Seoul 02841 Republic of Korea; ^4^ Center for Neuroscience Imaging Research Institute for Basic Science Suwon 16419 Republic of Korea; ^5^ Department of Biological Sciences Korea Advanced Institute of Science and Technology (KAIST) Daejeon 34141 Republic of Korea; ^6^ Creative Research Center for Brain Science Korea Institute of Science and Technology (KIST) Seoul 02792 Republic of Korea; ^7^ Department of Biomedical Engineering Sungkyunkwan University Suwon 16419 Republic of Korea; ^8^ Department of Intelligent Precision Healthcare Convergence Sungkyunkwan University Suwon 16419 Republic of Korea; ^9^ KAIST Institute for Health Science and Technology (KIHST) Daejeon 34141 Republic of Korea

**Keywords:** closed‐loop systems, preclinical studies, rapid eye movement (REM) sleep modulation, spatial working memory, therapeutics, ultrasound stimulation

## Abstract

Transcranial focused ultrasound stimulation (tFUS) is an effective noninvasive treatment modality for brain disorders with high clinical potential. However, the therapeutic effects of ultrasound neuromodulation are not widely explored due to limitations in preclinical systems. The current preclinical studies are head‐fixed, anesthesia‐dependent, and acute, limiting clinical translatability. Here, this work reports a general‐purpose ultrasound neuromodulation system for chronic, closed‐loop preclinical studies in freely behaving rodents. This work uses microelectromechanical systems (MEMS) technology to design and fabricate a small and lightweight transducer capable of artifact‐free stimulation and simultaneous neural recording. Using the general‐purpose system, it can be observed that state‐dependent ultrasound neuromodulation of the prefrontal cortex increases rapid eye movement (REM) sleep and protects spatial working memory to REM sleep deprivation. The system will allow explorative studies in brain disease therapeutics and neuromodulation using ultrasound stimulation for widespread clinical adoption.

## Graphical abstract

This work demonstrates a chronic, artifact‐free, and closed‐loop ultrasound stimulation system based on a capacitive micromachined ultrasound transducer for neuromodulation of the medial prefrontal cortex in mice. Stimulation during non‐rapid eye movement (NREM) sleep modulates REM sleep characteristics and short‐term spatial working memory. Neuroprotective effects of REM sleep are observed, which negates the adverse effects of REM sleep deprivation in spatial working memory.

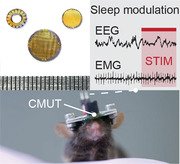

## 1 Introduction

Transcranial focused ultrasound stimulation is a promising therapeutic alternative to conventional pharmacological and other direct brain stimulation modalities because of its noninvasiveness, high spatial specificity, and long penetration depth.^[^ ^1^, ^2^, ^3^, ^4^ ^]^ To achieve clinical adoption, recent reports have explored the accuracy, efficacy, and safety of ultrasound stimulation via beam modeling simulations, magnetic resonance (MR) guided treatments, high spatial resolution systems, and histological safety studies in preclinical and clinical trials.^[^ ^5^, ^6^, ^7^, ^8^, ^9^, ^10^, ^11^, ^12^, ^13^ ^]^ These previous works have advanced the field toward expansive studies that explore the therapeutic effects of ultrasound stimulation in neurological and metabolic disorders such as Alzheimer's disease (AD), Parkinson's disease (PD), epilepsy, vascular stroke, inflammatory arthritis, obesity, and bone fracture healing.^[^ ^14^, ^15^, ^16^, ^17^, ^18^, ^19^, ^20^, ^21^, ^22^, ^23^, ^24^, ^25^ ^]^ However, these exploratory studies on the effects of ultrasound neuromodulation remain limited in neuroscience applications and widespread clinical translation due to the lack of general‐purpose preclinical systems for chronic stimulation in freely behaving animals.

Currently, there is no ultrasound neuromodulation system capable of long‐term stimulation and real‐time, closed‐loop neural recording in freely behaving animals. Existing technologies for noninvasive, chronic ultrasound stimulation in animals are anesthesia‐dependent, acute, or unable to simultaneously monitor neural recording during stimulation.^[^ ^26^, ^27^, ^28^, ^29^ ^]^ For instance, commonly used piezoelectric lead zirconium titanate (PZT) based transducers are too bulky for chronic freely moving studies in small animals and thus require head‐fixation under anesthesia or are limited to acute neuromodulation.^[^ ^30^, ^31^ ^]^ In addition, miniaturized PZT‐based transducers generate electrical artifact and are unsuitable for simultaneous electrophysiological recording in closed‐loop systems. Recent research on real‐time closed‐loop systems for rodents are also limited to head‐fixed, anesthesia‐dependent systems due to bulky transducers.^[^ ^32^, ^33^ ^]^ In larger animals such as non‐human primates (NHP), there have been several reports of closed‐loop systems with neuronavigational tools and behavioral modulation.^[^ ^34^, ^35^, ^36^ ^]^ However, these systems currently do not allow for stimulation during freely moving behaviors, and there have been no translational miniaturized systems for conducting similarly extensive studies in small animals. Consequently, long‐term preclinical studies observe the effects of ultrasound stimulation only after a sonication session under anesthesia.^[^ ^15^, ^16^, ^17^, ^19^, ^21^, ^37^ ^]^ This hinders the ability to observe natural behavior and monitor critical biosignals in animals, which restricts the range of clinical studies required to fully investigate therapeutic effects. Thus, while ultrasound neuromodulation has been gradually gaining acceptance as a clinical treatment modality, the potential of ultrasound treatment is limited by the lack of a general‐purpose stimulation system for preclinical experiments.

Here, we report a general‐purpose ultrasound neuromodulation system for chronic, closed‐loop preclinical studies in freely behaving rodents. We used microelectromechanical systems (MEMS) technology to design and fabricate a small and lightweight transducer capable of artifact‐free stimulation and simultaneous neural recording. The advantages of using silicon MEMS technology include high reproducibility and reliability, high precision manufacturing, and low electrical and MR artifacts. The MEMS transducer, named capacitive micromachined ultrasound transducer (CMUT), is electrostatically actuated and offers competitive advantages such as high design flexibility, miniaturization, and biomonitoring integration. The CMUT disk is designed to surgically integrate with electroencephalogram (EEG) electrodes for noninvasive, artifact‐free, and real‐time recording of brain activity. EEG is widely used as a clinical diagnostic tool for sleep disorders, epilepsy, and cognitive function.^[^ ^38^ ^]^ To demonstrate the potential of this closed‐loop, chronic, and artifact‐free system, we investigated the effects of ultrasound stimulation on natural sleep states across a span of three consecutive days without anesthesia. In addition, we explored the therapeutic effects of ultrasound stimulation on the spatial working memory of sleep‐deprived (SD) mice. We targeted the medial prefrontal cortex (mPFC), a crucial brain region in sleep that is linked to memory consolidation, dream formation, and REM sleep.^[^ ^39^, ^40^, ^41^ ^]^ Thus, the ability of our system to deliver stimulation in awake, naturally behaving mice enables long‐term, state‐dependent, and closed‐loop neuromodulation, which are essential in identifying therapeutic effects and developing treatment protocols.

## 2 Results

### 2.1 Design, Fabrication, and Packaging of Disk‐Type CMUT

**Figure 1:**
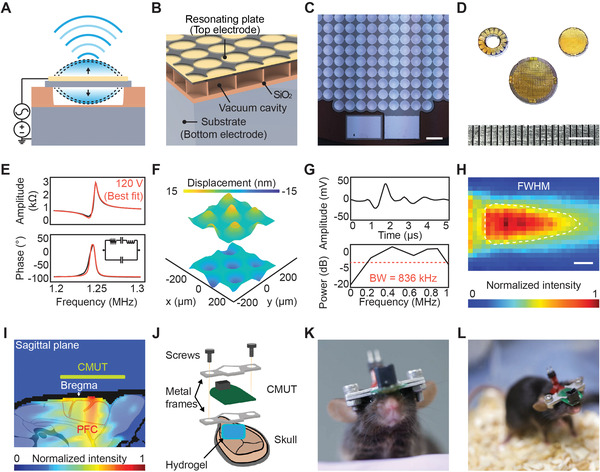
Design, fabrication, and in vivo packaging of CMUT device. A) Cross‐sectional schematic of a single CMUT cell consisting of an electrostatically actuated silicon plate. B) 3D cross‐sectional schematic of the CMUT device composed of multiple cells. C) Microscopic image of the disk‐type CMUT device (top view). Scale bar, 400 µm. D) Photo of various CMUT designs fabricated on a single silicon wafer. Top left: small ring CMUT, top right: small disk CMUT, bottom: large disk CMUT. Scale bar, 4 mm. E) Input impedance of the CMUT at 120 V DC bias voltage. The data was fitted to a Butterworth‐Van Dyke equivalent circuit model. F) Displacements of the CMUT plate visualized using a laser Doppler vibrometer (LDV). Uniform, in‐phase oscillation of seven cells was recorded over a 200 × 200 nm area. Color bar represents the displacement of the cells. G) Output pressure wave and corresponding frequency domain analysis of the large disk CMUT in immersion actuated with a 10‐V_pp_ 1‐µs pulse. Bandwidth of the CMUT is approximately 836 kHz. H) Beam profile of the large disk CMUT measured at the immersion resonant frequency, not accounting for skull effects. White dotted line indicates full width at half maximum beam size. Color bar indicates normalized intensity values. Scale bar, 700 µm. I) Simulated beam profile through a mouse skull model. Green line indicates CMUT location. White arrow represents bregma location. Color bar indicates normalized intensity values. J) Schematic of CMUT implantation apparatus. A surgically attached bottom metal frame and a removal top metal frame hold the CMUT in a tight seal on the skull. K,L) Photos of fully packaged CMUT devices surgically integrated with electroencephalography/electromyography electrode implants in freely behaving mice.

We designed a novel disk‐type CMUT device for chronic, closed‐loop in vivo ultrasound stimulation of the mPFC. A CMUT is a MEMS‐based ultrasound transducer, which consists of multiple micron‐scale capacitor cells all connected in parallel.^[^ ^26^, ^42^ ^]^ The top plates of the capacitor cells are actuated at its mechanical resonance through electrostatic transduction which generates and transmits ultrasound to the medium (**Figure** **1**A,B). Our disk‐type CMUT consisted of a total of 384 individual cells across a diameter of 8.1 mm (Figure S1, Supporting Information). To achieve a resonant frequency of 460 kHz (immersion) and an ultrasound intensity of 150 mW cm^−2^ (for 100 V_DC_ ± 100 V_pp_), we designed the cell radius, top silicon plate thickness, and vacuum gap height to be 90, 3, and 1.75 µm, respectively, using analytical methods, which were confirmed through numerical simulation (COMSOL Multiphysics, COMSOL Inc., Stockholm, Sweden) (Figure S1, Supporting Information).^[^ ^43^ ^]^

We successfully fabricated CMUT devices on a wafer level with high yield using direct wafer bonding process (Figure 1C,D, Figures S1 and S2, Supporting Information; Experimental Section). Each cell of the device was composed of a gold (Au) upper electrode, a silicon (Si) plate, a sealed vacuum cavity supported by silicon dioxide (SiO_2_) insulating posts, a SiO_2_ insulating layer, and a Si bulk substrate layer (Figure 1B). The gap was vacuum‐sealed to maximize power transmittance and reception sensitivity. A robust PDMS‐parylene C bilayer passivation method was used to encapsulate the packaged device for chronic applications (Figure S1, Supporting Information and Experimental Section).

### 2.2 Mechanical, Electrical, Acoustic, and Thermal Characterization of CMUTs

Next, we characterized the physical, electrical, acoustic, and thermal characteristics of the CMUTs. To confirm the physical structure of the fabricated disk CMUT, we visualized the cross‐sectional images of a single cell using a scanning electron microscope (SEM, SU5000, Hitachi, Japan) and the vacuum‐induced static deflection of the device with a 3D optical profiler (ContourGT, Bruker, MA, USA). The SEM image visualized a Si plate thickness of 2.95 µm and a SiO_2_ post height of 1.74 µm with a uniform gap, which was achieved by wafer‐level fusion bonding (Figure S1, Supporting Information). The deflection depth of the Si plate was approximately 800 nm and relatively uniform across the device, with the standard deviation of multiple adjacent cells less than a nanometer (Figure S1, Supporting Information). This confirmed the vacuum‐induced static deflection of the Si plate, which is critical for mechanical spring softening and transduction efficiency.

For evaluation of dynamic characteristics, the CMUT device was electrostatically actuated by applying both DC and AC voltages. At the mechanical resonant frequency, the AC voltage resonates the Si plate at its maximum displacement, while the DC bias voltage induces spring softening of the rigid plate to increase the transduction efficiency.^[^ ^44^ ^]^ At a DC bias voltage of 120 V in air, the mechanical resonance was at 1.24 MHz and the anti‐resonance was at 1.25 MHz (Figure 1E). We fitted our experimental data to the Butterworth–Van Dyke (BVD) equivalent circuit model and estimated the component values for the motional branch (Lx, Cx, and Rx) and static branch (C_0_). The Lx, Cx, Rx, and *C*_0_ values were 6.1 mH, 1.5 pF, 0.42 kΩ, and 265 pF, respectively (Figure S3, Supporting Information). As the DC bias voltage increased, the resonant frequency decreased due to the spring softening effect (Figure S3, Supporting Information).^[^ ^44^ ^]^ For ultrasound neuromodulation, we applied a DC bias voltage of 100 V, approximately 80% of the pull‐in voltage of the CMUT.^[^ ^43^, ^44^ ^]^ We observed the movement of the Si plate using a laser Doppler vibrometer (LDV; OFV‐534, OFV‐2570, Polytec GmbH, Germany) by applying both AC and DC voltages (100 V_DC_ ± 100 V_pp_). The LDV measurements showed approximately ±15‐nm uniform, in‐phase displacement of seven individual cells in a 200 × 200 µm area (Figure 1F, Movie S1, Supporting Information).

To determine the resonance frequency and 3‐dB bandwidth of the device in immersion, the CMUT was actuated with a 1‐µs pulse at 10 V_pp_ and 100 V_DC_, and the pressure output was analyzed using the fast Fourier transform (FFT) (Figure 1G). The resultant bandwidth was approximately 82% of the resonance frequency (460 kHz in immersion). Then, at the immersion resonance frequency, the beam profile of the device was measured (Experimental Section). The peak pressure amplitude was 55 kPa and the estimated maximum center intensity was 100 mW cm^−2^ (calculated under homogeneous and real acoustic impedance conditions). In addition, we compared the beam profiles of two different designs. The small disk‐type CMUT exhibited a full width at half maximum (FWHM) beam size of 5.6 mm^2^ and a focal length of approximately 1.2 mm (Figure S4, Supporting Information). The large disk‐type CMUT exhibited a FWHM beam size of 16.8 mm^2^ and a focal length of approximately 4.2 mm (Figure 1H, Figure S4, Supporting Information). We chose the large disk‐type CMUT for this study due to its higher beam intensity and larger target area capable of stimulating the entire mPFC (Figure 1I, Figure S4, Supporting Information). To quantitatively measure the effects of the mouse skull on the beam, we placed an ex vivo half skull in front of the transducer face. The overall beam attenuation was less than 30% and the distortion in transmission due to the presence of the ex vivo skull was negligible (Figure S4, Supporting Information). The resulting intensity of the beam (84 mW cm^−2^) is sufficient to effectively elicit motor responses.^[^ ^7^, ^26^ ^]^

Heat map simulations were conducted to assess the thermal effects of the ultrasound beam on tissues. Multiphysics software was used to design a bioheat transfer model based on Pennes’ Bioheat Transfer equation in a water medium for a 460 kHz acoustic disk source at 137.7 kPa peak pressure, which was more than double the actual device output of 55 kPa (COMSOL Multiphysics, COMSOL Inc.) (Supporting Information).^[^ ^45^ ^]^ At the beam focus, the heat diffused radially with an increase in temperature of approximately 0.36 °C at 300 s. At 2 s, the maximum rise in temperature was less than 0.02 °C (Figure S4, Supporting Information). In addition, self‐heating at the surface of the transducer was measured using an infrared (IR) thermometer (62 MAX Mini Infrared Thermometer, Fluke Co., WA, USA). Over a period of 1 min of continuous operation, the temperature at the surface of the CMUT increased by 1.5 °C (Figure S4, Supporting Information). Considering that our simulation protocol delivers ultrasound for 2 s at 6‐s intervals, the heat produced by the CMUT is nonsignificant. The negligible amount of generated heat is consistent with published reports on low‐intensity ultrasound beams.^[^ ^7^, ^23^, ^45^, ^46^, ^47^ ^]^

### 2.3 General‐Purpose Closed‐Loop Ultrasound Neuromodulation System with Real‐Time Electrophysiological Monitoring

**Figure 2:**
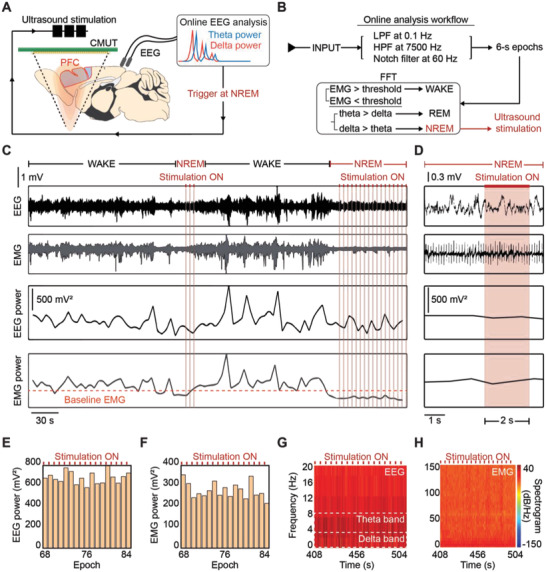
General‐purpose closed‐loop system with real‐time electrophysiological monitoring and artifact‐free ultrasound stimulation. A) Schematic of closed‐loop ultrasound stimulation and EEG monitoring system. An online biosignal analysis program triggers the ultrasound in real‐time during NREM sleep. B) Flowchart depicting the online EEG/EMG analysis program. C,D) Bandpass filtered EEG/EMG signals with power spectrum and sleep state analysis. Artifact‐free stimulation is shown across several minutes (C) and close‐up view of a single ultrasound trigger during NREM sleep shows no loss of signal integrity (D). E,F) Histogram of EEG power (E) and EMG power (F) for 17 consecutive epochs during NREM sleep stimulation. The red bars indicate 2‐s stimulation during each 6‐s epoch. No spike in power level is observed due to the stimulation. G,H) Spectrogram of EEG signal (G) and EMG signal (H) for 0–20 and 0–150 Hz, respectively. Signal power increases in the theta frequency band is observed as expected (G), and no large‐signal noise or artifact is present across the target frequency spectrum.

For simultaneous stimulation and electrophysiological monitoring in freely behaving mice, we developed a real‐time, closed‐loop ultrasound neuromodulation system. The CMUT device was packaged with electrophysiology electrodes on the mice skull for various behavioral experiments spanning four consecutive days (Figure 1J–L). Ultrasound stimulation was integrated with EEG and electromyography (EMG) electrodes, which are widely used biosignals for investigating neurological functions such as sleep (**Figure** **2**A). We used these electrodes to monitor natural sleep states without electrical artifacts during ultrasound stimulation. The complexity and importance of sleep as a crucial bioregulator served as an advantageous target for our general‐purpose artifact‐free, chronic, and natural‐state stimulation system. We developed a custom real‐time program (MATLAB, MathWorks, Natick, MA, USA) that analyzed incoming EEG and EMG signals in 6‐s epochs. The signals were digitally filtered with a 0.1 Hz low pass filter, 7.5 kHz high pass filter, and 60 Hz notch filter. We labeled each state WAKE (both active wakefulness and quiet wakefulness), REM, and non‐REM (NREM) based on the dominant power of the frequency bands for each state (Figure 2B, Experimental Section).^[^ ^48^, ^49^ ^]^ The triggers for the ultrasound stimulation were dynamically determined in real‐time based on the sleep state (e.g., NREM sleep initiation), which in turn was the outcome of previous neuromodulation cycles. Since the control action (i.e., trigger) is dependent on the output, we were able to achieve a closed‐loop neuromodulation system.

It is critical to maintain the integrity of neural signals during stimulation in order to apply real‐time, state‐dependent neuromodulation. We observed no artifacts during ultrasound stimulation in both EEG and EMG signals (Figure 2C–F). Moreover, we could deliver lengthy 2s long stimulation bursts for a 6 s long epoch duration without sacrificing signal integrity. A spectrogram of the signal power relative to the frequency spectrum also shows no high‐signal noise across 0–150 Hz, which spans the frequency bands for EEG and EMG (Figure 2G,H). Thus, using our system, it is possible to monitor and analyze the biosignals at the moment of stimulation, which is in contrast to clinically used electrical stimulation methods.

### 2.4 Ultrasound‐Induced Chronic REM Sleep Modulation During Natural Sleep

**Figure 3:**
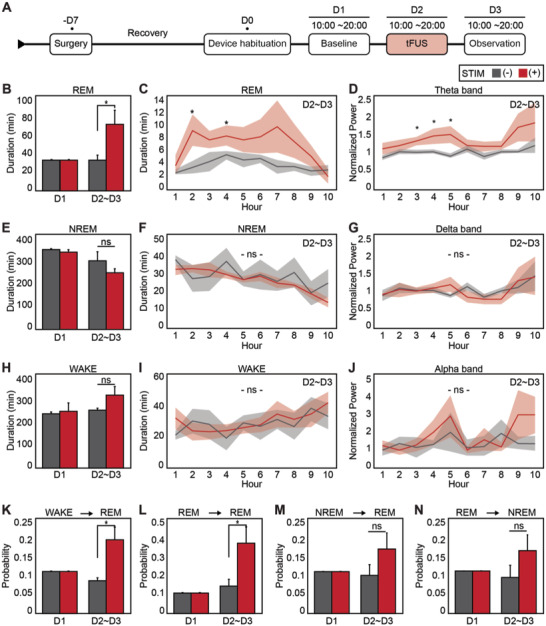
Modulation of sleep duration and sleep state transition. A) Experimental timeline for sleep modulation. Stimulation was delivered on day 2 (D2) over a 10‐h period. B–D) REM sleep analysis for total duration (B), hourly duration (C), and hourly theta frequency band power (D). The second (stimulation) and third days (observation) were averaged as D2–D3. The power spectrum was normalized to the baseline D1 levels. *p < 0.05, two‐sided unpaired Student's *t*‐test, *n = 20*. E–G) NREM sleep analysis for total duration (E), hourly duration (F), and hourly delta frequency band power (G). The power spectrum was normalized to the baseline D1 levels. ns, not statistically significant. H–J) WAKE state analysis for total duration (H), hourly duration (I), and hourly alpha frequency band power (J). The power spectrum was normalized to the baseline D1 levels. ns, not statistically significant. K–N) Probabilities of sleep state transition for REM sleep initiation from WAKE (K), REM maintenance (L), REM initiation from NREM (M), and NREM initiation from REM (N). *p < 0.05, two‐sided unpaired Student's *t*‐test, *n =* 20. ns, not statistically significant.

Using this artifact‐free, closed‐loop, and real‐time stimulation system, we conducted two in vivo experiments and investigated (1) sleep modulation via simultaneous stimulation and electrophysiological monitoring and (2) spatial working memory modulation in SD mice by observing spontaneous alternation behavior (SAB) in a Y‐maze. We stimulated during NREM sleep based on a pilot study conducted in anesthetized mice. In the pilot study, we observed a 14‐s acute increase in EEG delta power, which was analyzed offline following a 5‐min stimulation session of the mPFC (Figure S5, Supporting Information). The pulse train was constructed at a center frequency of 460 kHz, pulse repetition frequency (PRF) of 100 Hz, and duty cycle of 60% (Figure S5, Supporting Information). The CMUT device was inserted above the skull a day before baseline recording for 24‐h habituation (D0) (**Figure** **3**A, Experimental Section). Baseline measurements were acquired the next day (D1, 10:00–20:00) and ultrasound stimulation was delivered on day 2 (D2) for 10 h (10:00–20:00). On the third day (D3), observational recording was conducted to determine the chronic effects of ultrasound stimulation. Sleep duration, power spectrum, and stage transition probabilities for D2 and D3 were averaged as D2–D3.

We observed a significant increase in REM sleep duration for the stimulated mice (STIM(+)) on D2–D3 compared to the control group mice (STIM(−)) (Figure 3B; two‐tailed unpaired *t*‐test, *p < 0.05, *n* = 20). We further analyzed the sleep duration and frequency bands for D2–D3 by plotting the data per hour. For the 10‐h long period, REM sleep duration was increased for the STIM(+) mice across the entire period with statistically significant increases on the second and fourth hours (Figure 3C; two‐tailed unpaired *t*‐test, *p < 0.05, *n* = 20). The theta band power for the STIM(+) mice was also higher than the STIM(−) mice with statistically significant differences on the third, fourth, and fifth hours (Figure 3D, Figure S6, Supporting Information; two‐tailed unpaired *t*‐test, *p < 0.05, *n* = 20). On the other hand, there were no significant changes in the NREM sleep duration and delta power between the STIM(+) and STIM(−) mice (Figure 3E–G). In addition, no significant differences were observed in WAKE duration and alpha power between STIM(+) and STIM(−) mice (Figure 3H–J). We also calculated eight sleep state transition probabilities for REM, NREM, and WAKE. The probability of transition from WAKE to REM state increased significantly on D2–D3 for the STIM(+) mice compared to that of the STIM(−) mice, which resulted in a higher REM state initiation probability (Figure 3K; two‐tailed unpaired *t*‐test, *p < 0.05, *n* = 20). In addition, the probability of transition from REM to REM state also increased significantly on D2–D3 for the STIM(+) mice, which resulted in a higher REM maintenance probability (Figure 3L; two‐tailed unpaired *t*‐test, *p < 0.05, *n* = 20). However, the probability of transition from NREM to REM and REM to NREM increased nonsignificantly (Figure 3M,N; two‐tailed unpaired *t*‐test, *p* < 0.2, *n* = 20). The sleep state transition probabilities for the remaining four cases demonstrated no significant changes on D2–D3 (Figure S7, Supporting Information).

### 2.5 Ultrasound‐Induced Spatial Working Memory Modulation Using a Y‐Maze

**Figure 4:**
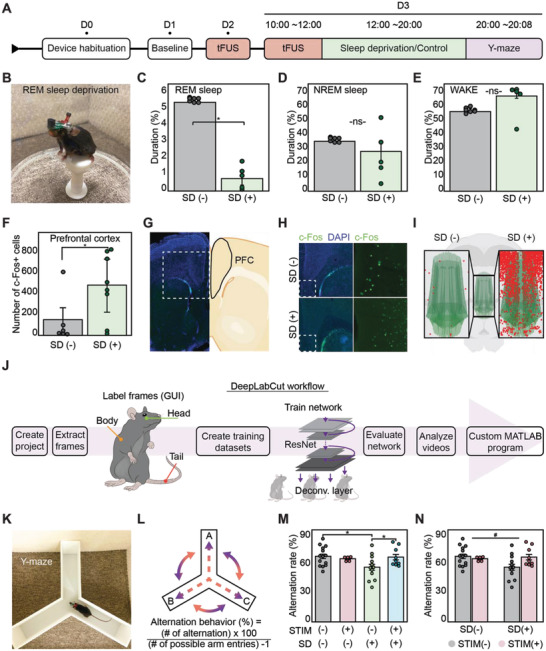
Modulation of spatial working memory via SAB in a Y‐maze. A) Experimental timeline for spatial working memory modulation via sleep deprivation and Y‐maze test. Acute SD was conducted over 8 h and Y‐maze SAB was observed for 8 min. B–E) REM SD using the small‐platforms‐over‐water method (B) was conducted and electrophysiological analysis of REM sleep (C), NREM sleep (D), and WAKE state (E) showed successful REM SD. *p < 0.05, two‐sided unpaired Student's *t*‐test, *n* = 7. ns, not statistically significant. F–I) c‐Fos IHC was conducted to verify REM SD. Total number of c‐Fos‐positive cells in the prefrontal cortex was significantly higher in the SD(+) mice (F). Whole brain section images (G) with DAPI staining (H) verified our cell counting method. Meshview was used to 3D‐map c‐Fos(+) cells in the entire prefrontal cortex (I). *p < 0.05, two‐sided unpaired Student's *t*‐test, *n* = 14. J) Workflow schematic for DeepLabCut, a semiautomated pose estimation program for animal behavior analysis. K,L) Photo of a mouse with the full neuromodulation system in Y‐maze (K) and schematic of the alternation rate calculation of spontaneous alternation behavior (SAB) (L). M) Spontaneous alternation rate in Y‐maze for SD(−) and SD(+) mice. N) Analysis of alternation rate with STIM and SD as factors. “*” represents comparison with STIM(−) SD(−) (*p* < 0.05, one‐way ANOVA with Tukey's post‐hoc test, *n* = 40), whereas “#” represents interaction between STIM and SD groups (*p* < 0.05, two‐way ANOVA with Tukey's post‐hoc test, *n* = 40).

To investigate the effects of ultrasound‐induced REM sleep modulation on spatial working memory, we conducted a memory test on REM SD mice. To induce REM SD, the small‐platforms‐over‐water method was implemented (**Figure** **4**A,B, Figure S8, Supporting Information, Experimental Section). Electrophysiological analysis showed that the small‐platforms‐over‐water method reduced REM sleep by 85.2% and NREM sleep by 26.9% (Figure 4C–E; two‐tailed unpaired *t*‐test, *p* < 0.05, *n = 7*). However, this method was reported to cause a significant elevation in stress level for chronic SD models.^[^ ^50^ ^]^ In order to quantify the stress level of mice during our acute 8‐h SD protocol, we sampled circulating corticosterone, a well‐established hormone for reporting bodily responses to environmental stress (Supplementary Information).^[^ ^51^ ^]^ The average post‐SD corticosterone levels were measured to be 212 and 285 ng mL^−1^ for the normal and SD mice, respectively (Figure S8, Supporting Information; two‐tailed unpaired *t*‐test, *p* = 0.234, *n = 6*). Thus, we believe that our 8‐h acute SD protocol caused minimal elevation in stress in mice. In addition, we attribute the high baseline corticosterone level in the mice to the cyclical nature of the hormone in synchronization with its circadian rhythm.^[^ ^51^ ^]^

In addition, to quantitatively measure the neuronal effects of our 8‐h REM SD on brain regions linked to sleep and memory, we conducted immunohistochemistry (IHC) of c‐Fos, an activity dependent immediate early gene that is widely used as a standard biomarker for neural activity.^[^ ^52^ ^]^ We analyzed the number of c‐Fos positive cells in the PFC, hypothalamic regions, and hippocampus, by visualizing 2D or 3D brain images using the QUINT workflow (Figure S8, Supporting Information).^[^ ^5^, ^53^, ^54^, ^55^ ^]^ The number of c‐Fos‐positive cells in the PFC, hypothalamus, and hippocampus increased significantly in the SD mice compared to the normal, non‐SD mice (Figure 4F–I, Figures S8 and S9, Supporting Information; two‐tailed unpaired *t*‐test, *p* < 0.05, *n* = 14). This was consistent with previous findings of elevated c‐Fos levels in SD animals.^[^ ^56^ ^]^ Our results confirmed that the method used for depriving sleep in this study reliably produced the physiological characteristics of sleep deprivation in mice.

Next, we investigated whether the observed REM sleep modulation affected the spatial working memory of mice. We could observe poststimulation behavior without the confounding effects of anesthetic intervention since our system was designed for chronic experiments in freely behaving animals. We chose the well‐established SAB test with a custom‐designed Y‐maze (NANUM Design, Daejeon, Korea) and an acute REM SD mouse model for both STIM(+) and STIM(−) groups (Experimental Section).^[^ ^57^ ^]^ We modified the experimental timeline to add an extra 2‐h stimulation period, an 8‐h SD protocol, and a Y‐maze SAB test on D3 (Figure 4A, Figure S8, Supporting Information). The SAB test was conducted at the end of the 8‐h SD/control period and the mice were gently placed inside the Y‐maze for 8 min. The mice were recorded using IR lighting and an HD webcam (Microsoft, WA, USA) in a soundproof sleep chamber (Sontek, Paju, Korea) (Figure S10, Supporting Information). The video data was analyzed offline using the widely used animal pose estimation program, DeepLabCut (DLC).^[^ ^58^ ^]^ Our DLC workflow labeled each frame from 40 videos and used the ResNet‐50 convolutional neural network (CNN) to train the dataset. With the mice trajectory data from DLC, a custom analysis program (MATLAB, Mathworks) was used to calculate the alternation rate (Figure 4J, Experimental Section). The Y‐maze alternation rate was calculated by counting each alternating‐triplet entry into the three arms (A to C) and dividing it by the total number of arm entries minus one (Figure 4K,L).^[^ ^57^ ^]^

The alternation rate of the STIM(−) SD(+) (54.858 ± 2.884; not stimulated, SD) mice was significantly lower than the STIM(−) SD(−) (66.173 ± 1.829; not stimulated, not SD) mice in the Y‐maze, which demonstrated a poorer spatial working memory due to the SD (Figure 4M and Figure S10, Supporting Information; one‐way analysis of variance (ANOVA) with Tukey's post‐hoc test, *F* = 5.158, *p < 0.05, *n* = 40; Movie S2, Supporting Information). This result was consistent with previous studies on sleep deprivation and memory.^[^ ^59^, ^60^ ^]^ In addition, there was a significant difference in the alternation rate between the STIM(−) SD(+) (54.858 ± 2.884) and STIM(+) SD(+) (66.615 ± 3.004) mice in the SAB test, which demonstrated that ultrasound stimulation protected the spatial working memory from the effects of sleep deprivation (Figure 4M and Figure S10, Supporting Information; one‐way ANOVA with Tukey's post‐hoc test, *F* = 5.158, *p < 0.05, *n* = 40; Movie S2, Supporting Information). Moreover, comparing the interaction between the STIM group and the SD group revealed a significant difference in the alternation rate, indicating that the stimulation effect on SAB is dependent on the SD condition (Figure 4N; two‐way ANOVA with Tukey's post‐hoc test, *F*_STIM_ = 2.517, *F*_SD_ = 2.042, *F*_STIM×SD_ = 6.784, ^#^ *p* < 0.05, *n* = 40). In other words, our data demonstrated that the effects of ultrasound stimulation were not apparent in the SAB for non‐SD mice.

We also analyzed the total number of arm entries for each experimental group, which demonstrated that sleep deprivation reduced the overall mobility of both the STIM(−) SD(+) (29.167 ± 4.205) and STIM(+) SD(+) (22.889 ± 2.627) mice compared to the STIM(−) SD(−) (43.643 ± 3.802) mice (Figure S10, Supporting Information; one‐way ANOVA with Tukey's post‐hoc test, *F* = 5.695, *p < 0.05, *n* = 40). Manual video analysis showed that the decreased mobility was due to an increase in grooming behavior while inside the Y‐maze. The increase in grooming behavior was attributed to the increase in short‐term anxiety experienced by the mice during the end of the sleep deprivation period and subsequent relocation into the Y‐maze.^[^ ^61^ ^]^ Nonetheless, this short‐term anxiety did not impair the spatial working memory of mice. There was also a significant difference in the number of arm entries between the STIM(+) (26.5 ± 2.353) and STIM(−) (36.962 ± 3.118) groups, which indicates the effect of ultrasound stimulation on overall mobility. Figure S10 (Supporting Information), one‐way ANOVA with Tukey's post‐hoc test, *F* = 5.166, *p < 0.05, *n* = 40). Two‐way ANOVA further revealed that both stimulation and SD significantly affected the number of arm entries (Figure S10, Supporting Information; two‐way ANOVA with Tukey's post‐hoc test, *F*_STIM_ = 3.92, *F*_SD_ = 8.277, *p* < 0.05, *n* = 40). In addition, we compared the DLC‐analyzed results with a manual visual analysis and found an error margin of approximately 3%, which indicated that our automated analysis setup was suitable (Figure S10, Supporting Information).

### 2.6 MR Compatibility of the General‐Purpose Ultrasound Neuromodulation System

**Figure 5:**
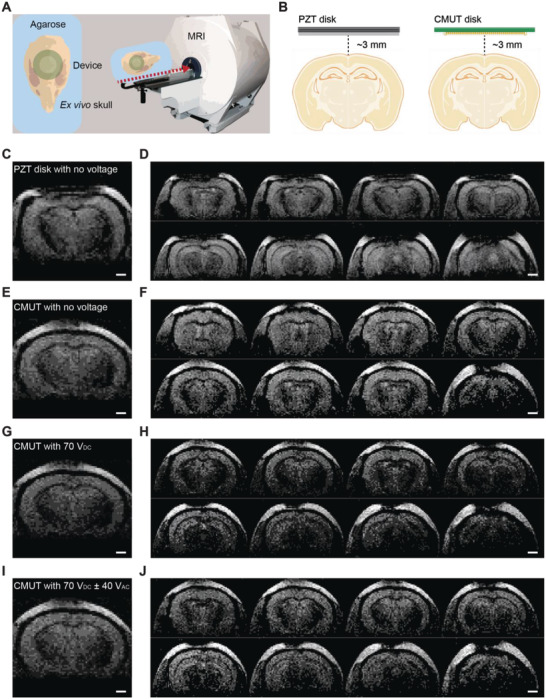
MR compatibility of the CMUT device using an ex vivo brain phantom. A) Schematic of agarose‐filled ex vivo mouse brain phantom and device inserted into MR imaging. B) Schematic of PZT disk and CMUT disk positioning above the brain phantom. C,D) EPI of brain phantom with the PZT disk (no voltage applied). C) Image signal of the brain below the PZT disk was significantly affected (D). E,F) EPI of brain phantom with the CMUT disk (no voltage applied). E) Brain images were not visibly affected by the CMUT (F). G,H) EPI of brain phantom with the CMUT biased at 70 V_DC_. G) Brain images were slightly affected but brain anatomy was identifiable (H). I,J) EPI of brain phantom with the CMUT driven at 70 V_DC_ ± 40 V_AC_ (I). Brain anatomy was clearly identifiable (J).

One critical disadvantage of noninvasive brain stimulation is not being able to visualize or confirm the stimulation site with respect to brain anatomy. To overcome this uncertainty, MR‐guided ultrasound has been widely used, especially for high‐intensity ultrasound tissue ablation used in clinical settings.^[^ ^62^ ^]^ In addition, the unrivaled benefits of MR imaging (MRI) for functional whole brain visualization, especially for large animals, has established MRI as a fundamental clinical tool.^[^ ^63^ ^]^ Thus, we demonstrated the MR compatibility of our MEMS‐based system by conducting MR echo planar imaging (EPI) in an ex vivo mouse brain phantom (**Figure** **5**A, Supporting Information). While PZT‐based transducers have been used in conjunction with MRI, PZT‐based transducers need to be placed far from the brain to be compatible with MRI.^[^ ^64^ ^]^ In contrast, because the CMUT device is composed of Type II MR‐compatible materials with magnetic susceptibilities close to water, the device can be placed near the brain without image distortion, which implies that the same setup used to validate the effects of ultrasound neuromodulation can be directly applied.^[^ ^65^, ^66^ ^]^

While the full demonstration of our system as “MR safe” (according to the American Society for Testing and Materials (ASTM) International) is beyond the scope of this work, here we show MR compatibility of our system compared to that of a conventional PZT4 ceramic disk using high‐resolution fast‐scanning EPI. EPI is particularly attractive for blood‐oxygen‐level‐dependent (BOLD) functional MRI (fMRI) studies, which are widely used for whole brain investigations of neural networks and are crucial for clinical applications.^[^ ^67^, ^68^, ^69^ ^]^ We demonstrate the advantage of CMUTs by comparing EPI signals with a size‐comparable PZT4 disk (*f*_0_ = 2 MHz, diameter = 8 mm, PZT Electronic Ceramic Co., Ltd, Guangdong, China) positioned at the same dorsoventral height above an ex vivo skull (Figure 5B). The EPI signal due to the PZT disk is significantly obscured and brain regions are not visible (Figure 5C,D). On the other hand, the CMUT disk does not cause major RF wave interference and the brain regions are clearly identifiable (Figure 5E,F). Furthermore, fully driving the CMUT with a 70 V_DC_ and ±40 V_AC_ has a negligible effect on the EPI signals (Figure 5G–J). While more work is needed for full verification of MR safety, the results indicate our preclinical system to be MR compatible, which further narrows the gap toward clinical translatability of ultrasound therapeutics.

## 3 Discussion

In this work, we demonstrate the design, fabrication, packaging, characterization, and application of a general‐purpose MEMS‐based ultrasound stimulation system in rodents. The design of the system was motivated by the need for a chronic, closed‐loop, and electrophysiology‐integrated ultrasound stimulator for naturally behaving animals. In rodents, in which a significant portion of preclinical work is conducted, the challenge was to fabricate a miniaturized device with robust packaging and artifact‐free integration with generic electrophysiological recording systems. The use of silicon‐based MEMS technology enabled fabrication of small, lightweight, and low‐noise CMUT devices, which were surgically integrated with EEG monitoring electrodes. The design parameters of the CMUT device were calculated using numerical analysis and finite element method simulations. Considerations for the ultrasound beam size and intensity were factored into the design, and several variations were fabricated on a single wafer. The use of standard silicon micromachining methods was critical for design flexibility. By modifying the cell radius and plate thickness of the CMUT, the resonance frequency can be readily altered to suit various biomedical applications (Equation S1, Supporting Information). Furthermore, the large bandwidth of our device (approximately 200–1000 kHz) enables a wide frequency spectrum for targeted ultrasound stimulation.

Because the proposed preclinical system allows for closed‐loop neuromodulation in naturally behaving rodents over a 10‐h period without intermittent anesthetic intervention, we were able to evaluate the effects of ultrasound neuromodulation on sleep and working memory. Sleep is a crucial and complex behavior manifest in all mammals and stimulation during natural sleep is critical for investigating brain circuits.^[^ ^70^, ^71^, ^72^, ^73^, ^74^, ^75^ ^]^ Ultrasound stimulation of the prefrontal cortex over a 10‐h period in mice shows an increase in REM sleep duration and REM sleep protection from acute sleep deprivation. The neurological link between the prefrontal cortex and REM sleep has been widely studied.^[^ ^39^, ^40^, ^41^ ^]^ However, we targeted a large region of the mPFC, which renders pinpointing an exact neural pathway a challenge. Future studies employing higher resolution ultrasound beams and neural recording tools could elucidate the neural pathways of the brain. In addition, this work is only a first demonstration of a general‐purpose ultrasound stimulation system for naturally behaving animals. An expansion of this work to various biological applications, such as treating sleep disturbances manifest in PD or AD, is needed to prepare for clinical translation. In conclusion, this research demonstrates the potential of MEMS‐based general‐purpose ultrasound neuromodulation systems for various chronic preclinical studies in naturally behaving rodents.

## 4 Experimental Section

### Study Design

designed, fabricated, and packaged a MEMS‐based ultrasound stimulation system for general‐purpose neuromodulation in chronic, freely behaving preclinical studies. This system was used to demonstrate artifact‐free stimulation and simultaneous EEG/EMG recording in mice for sleep and memory modulation. Further investigations of experimental design were conducted using corticosterone ELISA, c‐Fos IHC, and MR imaging.

### Animal Care and Study Approval

All animals used in this study were in accordance with protocols outlined and approved by the Institutional Animal Care and Use Committee at the Korea Advanced Institute of Science and Technology (KA2021‐066). A total of 87 male C57Bl/6J mice (6–8 weeks old) were used in the experiments. Animals were housed in separate cages in a sound‐proof sleep chamber (Sontek) under a 12‐h light/dark cycle (lights ON at 07:00) with ad libitum access to food and water.

### Microfabrication Process Flow

The disk CMUT devices were fabricated on a 4‐inch silicon wafer (Figure S2, Supporting Information). A highly doped silicon wafer was used as the bottom substrate layer, which served as the bottom electrode contact. A double oxidation process defined the 1.75‐µm vacuum cavity. An initial 2‐µm oxide layer was thermally grown on the silicon wafer at 1100 °C, then the cavities were etched using a photoresist mask layer and buffered oxide etch (BOE) 6:1 solution. The second oxide layer was thermally grown (1100 °C) at a thickness of around 250 nm to insulate the exposed bottom silicon layer. This patterned substrate was then bonded to a highly doped silicon‐on‐insulator (SOI) wafer through the direct wafer bonding method. The handling substrate and buried‐oxide (BOX) layer of the bonded SOI wafer were removed using chemical mechanical polishing (CMP) and BOE 6:1 wet etching process which exposed the 3‐µm silicon layer of the SOI bonded to the patterned substrate. Then, this top silicon plate was etched using reactive ion etching (RIE) to pattern the disk shape for the 384 cells. The oxide layer was etched using BOE 6:1 to access the bottom silicon substrate. Then, 10‐nm thick chromium (Cr) and 240‐nm thick gold (Au) layers were deposited and wet etched to pattern the electrical contacts with the top electrode and bottom substrate electrode layers. Finally, deep RIE was used to release each device from the whole wafer.

### CMUT Packaging

For the in vivo studies, the device was packaged on a custom‐designed printed circuit board (PCB) with gold microwires that were fixed with epoxy resin. The gold wires were bonded from the patterned signal and ground pads on the device to their respective metal connections on the PCB. Then a bilayer passivation coating was applied in two steps: (1) 0.5 mm PDMS 10:1 dip coating with a step speed of 1 mm s^−1^ and (2) 1 µm parylene C deposition (Young Hi‐Tech, Korea). The PDMS solution was degassed in a vacuum chamber for 30 min prior to the coating and there were no air pockets or bubbles in the coating layer. The PDMS layer insulated the top electrode layer of the device while the parylene C layer protected the soft PDMS layer from physical damage and minimally affected the beam transmission and mechanical resonant frequency. The bilayer passivation electrically insulated the device and improved the robustness of the device for chronic applications (Figure S1, Supporting Information). Lastly, to provide the electrical connection, the fully packaged device was wire‐bonded to a PCB integrated with micro‐pin connectors (Figure S1, Supporting Information). An external electrical connection was made by soldering a 2‐pin connector to the PCB edge, which was wired to a bias‐T circuit for AC and DC input. In addition, an impedance matching layer was not required because the silicon CMUT plates exhibit relatively lower acoustic impedance mismatch to biological tissues.^[^ ^76^ ^]^

### Surgical Procedure

Mice were anesthetized with isoflurane (4% induction, 1.5% maintenance) and fixed onto a stereotaxic frame (RWD Life Science Co., Ltd., China) for electrode implantation and frame attachment surgery. Ophthalmic ointment was applied on the eyes to prevent them from drying out, and lidocaine was injected in small amounts into the mouse scalp to reduce pain. The fur on top of the skull was gently removed, then the scalp was carefully excised to expose the skull. Medical‐grade 0.9 NaCl saline was used to clean the skull surface and two stainless steel screws for EEG recording were inserted halfway into the skull (signal, ML: 2, AP: −3 mm; and ground, ML: −2, AP: −5 mm). The EMG electrode was inserted into the nape (trapezius muscle) and the EEG ground electrode was used as the common electrical ground. The EEG/EMG electrodes were fixed to the skull using dental cement and any exposed conductive surfaces were covered (except for the output socket pins of the electrodes) in order to minimize noise during recording. Then, an aluminum metal frame (30 × 10 mm), which was the bottom frame for holding the CMUT in place during chronic stimulation, was attached to the skull above the prefrontal cortex (center ML: 0, AP: 2 mm) using Loctite adhesive (Figure 1J, Figure S11, Supporting Information). All mice for all experimental groups underwent the same surgical procedure and recovery timeline. Mice were given 7 days for recovery with food and water provided ad libitum.

### Electrical Impedance Characterization

The electrical input impedance of the disk CMUT was measured using an impedance analyzer (E4990A, Agilent Technologies Inc., CA, USA) and an attached probe station. A bias‐T circuit was used to superimpose an external high‐DC voltage (PS310, Stanford Research Systems Inc., Sunnyvale, CA, USA) and a 50‐mV AC voltage supplied internally from the impedance analyzer. The impedance magnitude and phase were measured over a frequency range of 100 kHz to 5 MHz for five DC voltages in 10‐V increments from 80 to 120 V (Figure S3, Supporting Information).

### Ultrasound Stimulation Setup

The equipment used to drive the CMUT consisted of two function generators (33220A, Agilent Technologies), a 47‐dB RF amplifier (5312F, OPHIR RF, CA, USA), and a high‐DC voltage supply (PS310, Stanford Research Systems Inc.). A bias‐T circuit, consisting of a resistor and DC‐filtering capacitor on a custom PCB, was used to combine the AC and DC inputs to actuate the CMUT. The first function generator defined the duty cycle and PRF of the trigger square wave, which was input to the second function generator. The second function generator generated the AC sine wave at the immersion resonant frequency of the CMUT, which was then amplified by the RF amplifier and delivered to the bias‐T circuit (Figure S5, Supporting Information). The resulting AC and DC signal from the bias‐T circuit was used to actuate the CMUT at the desired amplitude and frequency.

### Beam Profile Measurement

We measured the beam profile of the packaged CMUT using a custom‐made acrylic oil tank (Sciencetown Inc., Incheon, Korea) filled with degassed soybean oil. The oil tank was fitted with a programmable motorized stage (Sciencetown Inc.) capable of movement in the micron scale, and a needle hydrophone (NH0500, Precision Acoustics, UK) was attached to an arm of the motor. A custom‐made MATLAB program (Mathworks) was used to scan an 8 × 8 × 10 mm cubic volume centered at the focal spot of the ultrasound beam. The output of the hydrophone was amplified and DC coupled (Precision Acoustics), and the resultant signal was acquired by a digital oscilloscope (DSOX2022A, Agilent Technologies Inc.).

In addition, we extracted a mouse skull and carefully removed the ventral half of the skull such that only the dorsal, upper half remained. The brain was removed and the ex vivo half skull was placed between the focal spot of the ultrasound beam and the CMUT (Figure S4, Supporting Information). A beam profile was obtained using the same protocol as before in order to quantify the effects of the skull layer on beam attenuation and distortion.

### In Vivo Ultrasound Stimulation

On D0, a day before baseline EEG/EMG recording, a hydrogel coupling medium was applied on the exposed skull, and the CMUT device was inserted in between the bottom (implanted) metal frame and a top metal frame. The metal plates and device were fixed in place using a pair of stainless‐steel nuts and bolts. This procedure was subjected to both STIM(+) and STIM(−) groups, with a dummy device used for the STIM(−) mice. This setup allowed for chronic ultrasound stimulation in a freely moving setup (Figure S11, Supporting Information). We further verified that the hydrogel did not dry out for the 10‐h duration of the experiment (Figure S11, Supporting Information).

On D1, the baseline EEG/EMG recording was conducted for 10 h (10:00–20:00) and the thresholds for delta power, theta power, and EMG power were determined. Based on the threshold values and manual video analysis of 5 days, the baseline NREM, REM, and WAKE durations were set to 55%, 5.5%, and 39.5% respectively.^[^ ^77^ ^]^ On D2, 30 min before the start of the experiment, the mice were lightly anesthetized with 2% isoflurane and the hydrogel between the CMUT and skull was replenished for both STIM(+) and STIM(−) mice. The mice were returned to the chamber and observed until they completely regained normal, pre‐anesthesia motor function and behavior. The recovery time was approximately 10 min. Ultrasound stimulation was delivered to the STIM(+) mice, while the STIM(−) mice were not stimulated. On D3, the hydrogel was again replenished, but no stimulation was given for both STIM(+) and STIM(−) mice. Observational EEG/EMG recording was conducted on D3 to determine any chronic effects of ultrasound stimulation of the mPFC on sleep.

### EEG and EMG Recording and Real‐Time Processing

The EEG/EMG signals were sampled at a rate of 1 kHz and digitally amplified with a gain of 1000 using a biopotential acquisition device (RHD2000, Intan Technologies, CA, USA). The signal was then filtered with a 0.1 Hz low‐pass filter, 7.5 kHz high‐pass filter, and 60 Hz notch filter. A custom‐written MATLAB program (Mathworks,) was used to partition the signal into 6‐s epochs and run the FFT analysis for each incoming epoch of real‐time signal. For every epoch, the FFT analysis determined the sleep/wake state of the mice by comparing the EEG/EMG power spectrum of the signal in the frequency domain. EEG delta waves (0.5–4 Hz) are dominant during NREM sleep, theta waves (4–8 Hz) are dominant during REM sleep, and alpha waves (8–12 Hz) are dominant during WAKE states. In addition, the EMG power is greatly reduced during sleep states, particularly during REM sleep. The baseline recording and analysis conducted on D1 determined the thresholds for REM, NREM, and WAKE states. An epoch was identified as NREM sleep when the EEG delta power was greater than the threshold and was identified as REM when the EMG power and EEG theta power were lower than the threshold values. A WAKE epoch was determined when the EMG power was 1.7 times greater than the threshold value.^[^ ^48^, ^49^ ^]^ On D2, when the program detected a NREM epoch, the trigger for ultrasound stimulation was delivered.

### Y‐Maze Design and Spontaneous Alternation Behavior (SAB) Protocol

The Y‐maze used in this study was custom‐made using matte white acrylic with a height of 15 cm, arm length of 20 cm, and arm width of 7 cm (NANUM Design). The maze was large enough for the mice to turn around and travel along the length of the arm comfortably. We used a sound‐proof animal testing chamber to house and conduct all in vivo behavioral experiments (Sontek). The Y‐maze was used to test the spatial working memory of mice as it has been reported to be affected by REM SD and could be used as a simple behavioral marker for cognitive function. SAB has been widely observed to test spatial working memory without the need for complicated conditioning tasks and training. The SAB refers to the innate preference of mice for entering a previously unexplored arm of the Y‐maze. Due to the natural curiosity of the mice, the animals will alternate entry into the arms at an average alternation rate of approximately 60–70%. Each arm of the maze was labeled “A,” “B,” and “C,” and a successful alternation was counted for every triplet of arm entries where the mice explored all three different arms. The alternation rate or index, was calculated by dividing the number of total alternations by the total number of possible arm entries minus one.^[^ ^57^, ^78^ ^]^

### Development of a REM Sleep‐deprived Model

We chose the widely used small‐platforms‐over‐water method, also known as the flower pot method, for REM SD. The setup consisted of a custom‐designed clear acrylic cylindrical cage (NANUM Design) with 30‐cm high sidewalls, a platform at the center of the cage composed of a 5.2‐cm high plastic support structure, and a 3.1‐cm diameter falcon tube cap (Figure S8, Supporting Information).^[^ ^50^ ^]^ Warm water was filled at a height of 2 cm before the mice were gently placed on the platforms for SD. In order for the mice to stay on the platform without falling off, the platform was designed to be sufficiently large for the mice to groom and sufficiently small in size for the mice to lose balance at REM sleep initiation. In addition, the mice were habituated to handling by a single experimenter for 2 days prior to SD so that the mice were calm when placed on the SD platform. Food and water were provided ad libitum throughout the 8‐h SD period. Electrophysiological recording (EEG and EMG), corticosterone ELISA, and c‐Fos IHC verified successful REM SD (Figure 4, Figures S8 and S9, Supporting Information).

### DeepLabCut Analysis Setup

For analyzing the Y‐maze behavior, we used DLC, a well‐established program for animal pose estimation based on widely‐used machine learning (ML) algorithms.^[^ ^58^, ^79^ ^]^ We installed a graphics processing unit (GPU) based version of DLC on a Linux system running Ubuntu 18.04 equipped with a 32GB AMD Ryzen 7 1700 8‐core processor. Then, we trained a representative video from each of the four experimental groups: STIM(−) SD(−), STIM(−) SD(+), STIM(+) SD(−), and STIM(+) SD(+). The head, body, and tail of the mice were manually labeled for the four representative videos. Using the ResNet‐50 CNN, we trained the system for 110 000 iterations. Our trained DLC network automatically labeled every frame from a total of 40 videos and output the movement trajectory data for each mouse. Using the trajectory data, a custom‐made MATLAB program (Mathworks) calculated the spontaneous alternation rate by designating IN and OUT zones for each of the three arms of the Y‐maze. A successful entry into an arm was counted when the whole body was inside the arm and an exit from an arm was counted when the entire body left the center of the arm. In addition, we manually counted the SAB rate by visually analyzing all 40 videos in order to verify our DLC analysis results (Figure S10, Supporting Information).

### Statistical Analysis

Statistical analysis was performed on normally distributed data sets confirmed using Shapiro–Wilk's normality test. Parametric analysis was conducted using a two‐sided unpaired Student's *t*‐test (unequal variances) for electrophysiology and immunohistology data, and one‐way and two‐way analysis of variance (ANOVA) with Tukey's post‐hoc test for behavioral data. For one‐way ANOVA, experimental group was used as the factor and for two‐way ANOVA, experimental group (stimulation condition) and SD were used as factors.

All data were analyzed using Microsoft Excel (Microsoft), OriginPro 2019 (OriginLab Co., Northampton, MA, USA), and MATLAB R2020b (MathWorks). Unless stated otherwise, all data are presented as means ± standard error of means (s.e.m.) and a *p*‐value of <0.05 was considered significant.

## Conflict of Interest

The authors declare no conflict of interest.

## Author contributions

Y.J., H.K., S.K., and H.J.L. performed conceptualization; Y.J. and H.K. worked on methodology; Y.J., S.M.L., C.L., G.H.I., S.L., H.K., and S.K. performed investigation; Y.J., T.J., G.P., J.S.P., C.O., and G.K. worked on visualization; H.J.L. and J.K. did funding acquisition; H.J.L. worked on project administration; H.J.L. and J.K. supervised the work; Y.J. and H.J.L. wrote the original draft; Y.J., H.J.L., J.K., S.L., and G.S.B.S. reviewed and edited the writing.

## Supporting information

Supporting Information

Supplemental Movie 1

Supplemental Movie 2

Supplemental Movie 3

Supplemental Movie 4

Supplemental Movie 5

## Data Availability

The data that support the findings of this study are available in the supplementary material of this article.
