# Supplementary material for: General‐Purpose Ultrasound Neuromodulation System for Chronic, Closed‐Loop Preclinical Studies in Freely Behaving Rodents
Source: Adv Sci (Weinh). 2022 Oct 19;9(34):2202345. doi: 10.1002/advs.202202345 (PMC9731702; doi:10.1002/advs.202202345)
Supplement: Supplementary file 1 — Supporting Information [file ADVS-9-2202345-s004.pdf]

Supporting Information

General-purpose ultrasound neuromodulation system for chronic, closed-loop preclinical studies in freely behaving rodents

Yehhyun Jo, Sang-Mok Lee, Taesub Jung, Gijae Park, Chanhee Lee, Geun Ho Im, Seongju Lee, Jin Soo Park, Chaerin Oh, Geon Kook, Hyunggug Kim, Seongyeon Kim, Byung Chul Lee, Greg S.B. Suh, Seong-Gi Kim, Jeongyeon Kim, and Hyunjoo J. Lee**

Materials and Methods

Enzyme-linked immunosorbent assay (ELISA) for corticosterone measurement: To quantify the levels of stress caused by our acute 8-h SD, we conducted ELISA for circulating corticosterone. Blood samples were extracted from the tail vein of 6 mice (3 normal, 3 SD) at 10:00 and again after an 8-h SD (12:00 ~20:00) at 20:00. Each serum sample was then separated from the blood at 13,000 rpm at 4°C for 10 minutes by centrifugation. The serum was stored at 4°C overnight for analysis the next day. Corticosterone concentrations were determined using a commercial ELISA kit (RE52211, IBL international GmbH, Hamburg, Germany). All samples were conducted in triplicate and the absorbance was measured at 450 nm using a plate reader (Infinite M200, Tecan). The results were calculated automatically using a 4-parameter curve fit (Figure S8).

Immunohistochemistry protocol: Immediately following an acute 8-h sleep deprivation session, mice were anesthetized with isoflurane (induction at 3%) and 2% Avertin (20 µl/g, i.p.), then transcardially perfused with 4% paraformaldehyde in phosphate buffered saline (PBS) (Figure S8). The brains were extracted and post-fixed in 4% paraformaldehyde at 4°C for 24 hours

before washing with PBS and incubating in a 30% sucrose solution at 4°C until they shrank for cryoprotection. Coronal, 50-μm thick brain slices were obtained using a cryostat microtome (Leica).

Brain slices were incubated with blocking and permeabilization solution (PBS containing 0.2% triton X-100, 0.5% BSA and 4% normal goat serum) for 1h 30 min at room temperature, and then incubated overnight with primary antibody (rabbit anti-cFos, cell signaling technology, 1:1000) in blocking solution (0.5% BSA with 4% normal goat serum) at 4°C. Fluorescent secondary antibody (goat anti-rabbit Alexafluo-488, Invitrogen, 1:500) were treated at room temperature for 1 h 30 min. The sample was washed in PBS three times and then mounted on slide glasses (Thermo Scientific) with DAPI containing a mounting medium (Vectashield). A slide scanner (Pannoramic SCANII, 3DHISTECH Ltd., Hungary) was used to obtain fluorescent images of the brain slices and QUINT workflow was used to analyze the images. The QUINT workflow was used in a previous paper, and consisted of Nutil, QuickNII, ilastik software, and a custom-made MATLAB program for cell counting (Figure S8).[5] First, c-Fos-positive cells were identified using Ilastik and Image J software.[53] Then, the fluorescent images were aligned to a standard mouse brain atlas using QuickNII program.[55] Lastly, Nutil Quantifier was used to analyze the images and count the number of c-Fos-positive cells in target brain regions.[54] A custom MATLAB program parsed the quantified data and graphically represented the c-Fos-positive brain regions (Figure S8).[5]

Ultrasound beam simulations in a mouse skull model: To confirm beam transmission through the skull and accurate targeting of the medial prefrontal cortex (mPFC) of mice, we conducted acoustic simulations using the k-space pseudo-spectral method and the open source MATLAB k-

Wave acoustics toolbox (Mathworks, Natick, MA, USA). We obtained a 3D model of a mouse cranium CT data from *Thingiverse*, which was originally provided by Dr. Mark Henkelman of the Mouse Imaging Center (MICe) in Canada. The parameters of the acoustic wave propagation were as follows. The speed of sound was 1500 $m \cdot s^{-1}$ for water and 3360 $m \cdot s^{-1}$ for the mouse cranium. The density of the medium was 1000 $kg \cdot m^{-3}$ for water and 1600 $kg \cdot m^{-3}$ for the skull. The acoustic attenuation coefficient was 0.0022 for water and 2.36 for the skull. A spatial step of 50 μm and temporal step of 4.4643 ns were used for the simulation space. (Courant-Friedriches-Lewy number (CFL) of 0.134 in water and spatial sampling of 6 grid points per wavelength in water for a frequency of 5 MHz).[5]

Thermal evaluation of ultrasound beam: Ultrasound beam simulations were conducted using COMSOL Multiphysics software to determine heat transfer. An 8-mm transducer disk was modelled as the acoustic source with a 460 kHz resonance frequency and 15-nm displacement resulting in a 137.7 kPa beam focus. The bioheat model, based on Pennes' Bioheat Transfer equation, was generated in a water medium with an absorption coefficient value of 0.025 m^{-1} and initial temperature of 37 ºC. For a conservative estimation, external cooling factors and blood perfusion rate were not considered. The beam was recorded for 300 s, which is 150 times longer than the 2-s *in vivo* stimulation pulse given in this study.

Magnetic resonance imaging (MRI) with ex vivo mouse brain phantom: Magnetic resonance imaging (MRI) was conducted to confirm CMUT compatibility with MR imaging techniques. A fully-packaged and PCB-integrated disk-type CMUT was fixed on an *ex vivo* mouse brain phantom for MR imaging. Using this setup, we acquired images for the following parameters: (1)

CMUT with no electrical input, (2) CMUT with a DC bias of 70 V, and (3) CMUT under a 70 V_{DC} ± 40 V_{AC} operation. In addition, a 2 MHz PZT disk (diameter = 8 mm, PZT Electronic Ceramic Co., Ltd, Guangdong, China) was packaged using a PDMS passivation layer and fixed on the brain phantom at the same height as the CMUT. A conventional low-pass filter (f_c = 2.5 MHz) was used to filter out high frequency noise from the RF amplifier.

MR imaging experiments were conducted in a horizontal bore 9.4 T/30 cm Bruker BioSpec MR system (Billerica, MA, USA) with an actively shielded insert (d = 12 cm) operating at a maximum strength of 66 gauss/cm and a rise time of 141 µs. A quadrature birdcage coil (d = 86 mm) was used for excitation, and an actively decoupled planar surface coil (d = 10 mm) was positioned on top of the brain phantom. The CMUT device was placed within the inner diameter of the surface coil. Using a field map method, the magnetic field homogeneity was shimmed, first globally then optimized using the MAPSHIM protocol with a shim volume covering the cerebrum (ParaVision 6, Bruker BioSpin). Single-shot gradient echo (GE) echo planar imaging (EPI) was conducted with the following sequence parameters: sampling frequency = 217 kHz, TR/TE = 1000/15 ms, flip angle = 54°, number of averages = 1, field of view = 15 (readout) × 7.5 (phase encoding) mm^2, matrix = 96 × 48, in-phase resolution = 156 × 156 μm^2, and slice thickness = 500 µm.[68, 69]

Supplementary Equation

$$f_0 = \frac{2t}{\pi a^2}\sqrt{\frac{5E}{9\rho(1-\upsilon^2)}}$$

Equation S1. Fundamental resonance frequency of a circular vibrating plate. The t is the thickness of a circular cell, a is the radius of the cell, E is the Young's modulus of silicon, ρ is the density of silicon, and υ is the Poisson's ratio.

Supplementary Figures

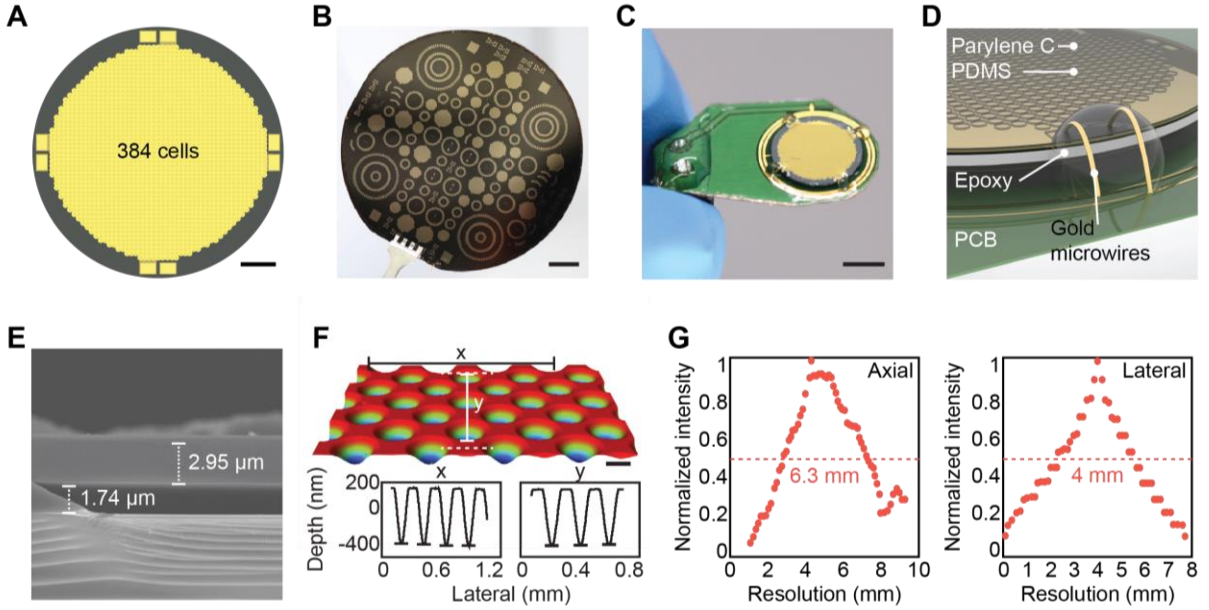

Figure S1. Fabrication design, mechanical characteristics, and packaging of CMUT device. (**A**) Schematic of a disk-type CMUT device consisting of 384 parallel-connected cells. Scale bar, 0.7 mm. (**B**) Photo of a silicon wafer consisting of various fabricated CMUT designs. Scale bar, 1 cm. (**C**) Photo of fully-packaged CMUT device. Scale bar, 4 mm. (**D**) Schematic of bilayer passivation and packaging of CMUT device. (**E**) Cross-sectional SEM image of a fabricated CMUT cell. The top silicon plate thickness is approximately 2.95 µm and the vacuum gap is approximately 1.74 µm. (**F**) 3D optical profile of static plate deflection of a CMUT device. Scale bar, 90 µm. (**G**) Beam profile of the CMUT measured at the immersion resonant frequency. The FWHM axial and lateral resolutions are 6.3 mm and 4 mm respectively.

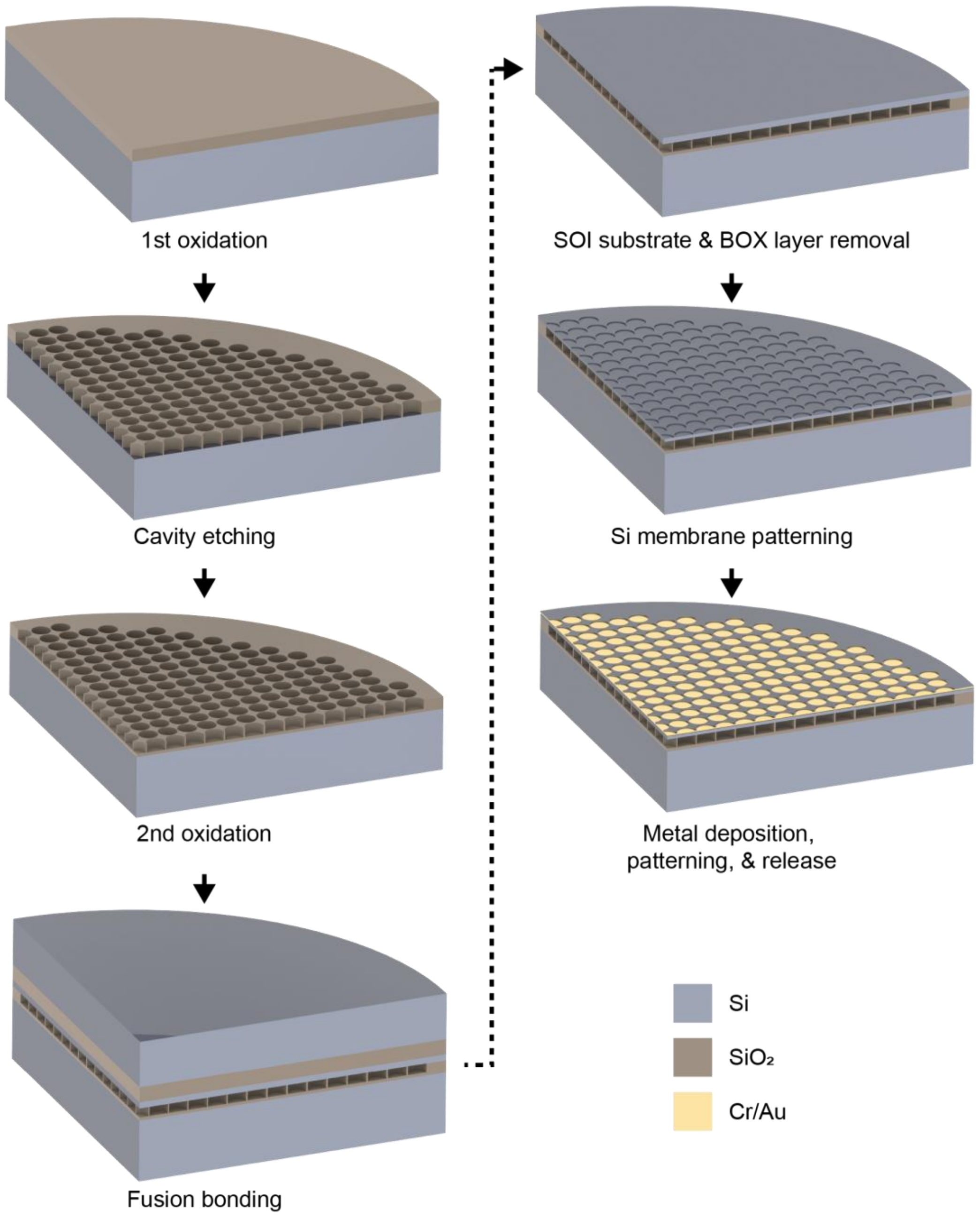

Figure S2. Wafer-level fabrication process of CMUT devices using direct wafer-bonding process.

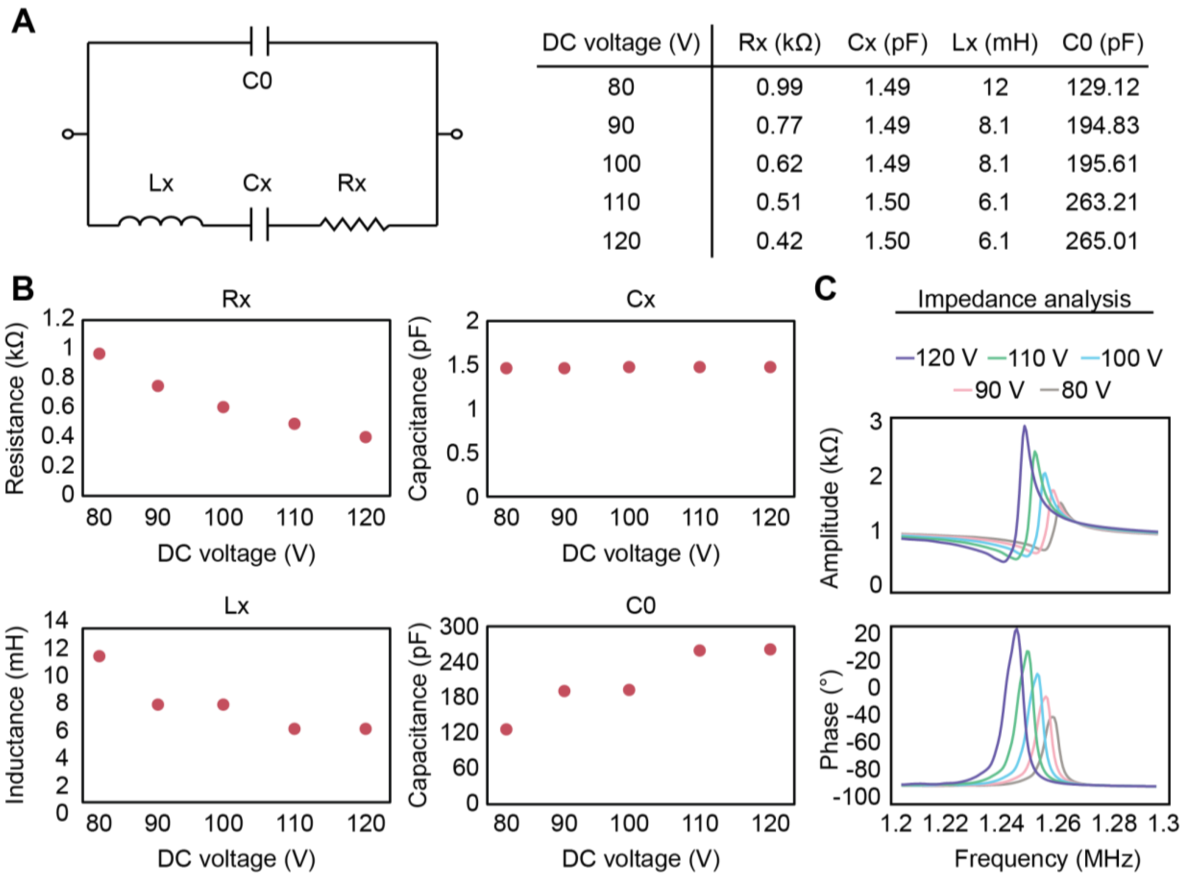

DC voltage (V)	Rx (kΩ)	Cx (pF)	Lx (mH)	C0 (pF)
80	0.99	1.49	12	129.12
90	0.77	1.49	8.1	194.83
100	0.62	1.49	8.1	195.61
110	0.51	1.50	6.1	263.21
120	0.42	1.50	6.1	265.01

Figure S3. Impedance analysis using Butterworth-Van Dyke (BVD) equivalent circuit model. (**A**) Schematic of BVD equivalent circuit and component values for five DC bias voltages. (**B**) Values of BVD circuit components for five DC bias voltages. (**C**) Input impedance of CMUT for five DC bias voltages.

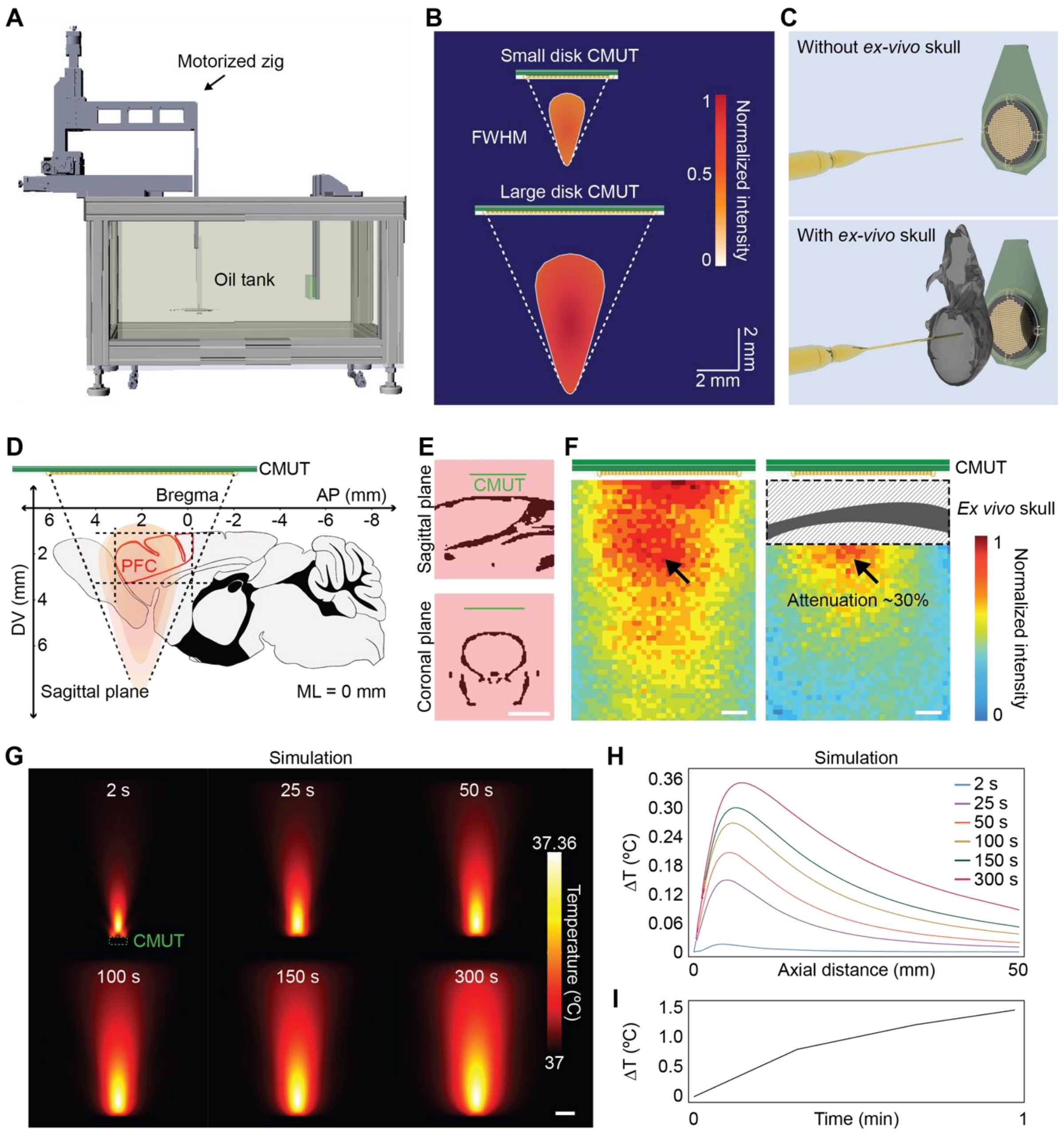

Figure. S4. Beam profile measurement. (**A**) Schematic of a tank with a motorized xyz zig and a hydrophone to measure immersion beam profile. (**B**) Beam profiles for small disk CMUT and large disk CMUT. Recolored using Adobe Illustrator for normalizing intensity for both beams. The color bar represents normalized intensity. (**C**) Schematic of beam measurement setup consisting of the CMUT and hydrophone with and without *ex vivo* mouse skull. (**D**) Schematic of ultrasound beam focus relative to the mouse brain and prefrontal cortex target region. Landmark

anatomical coordinates such as the bregma are indicated. (**E**) Mouse skull simulation schematic depicting CMUT location relative to the skull in the sagittal and coronal planes. Scale bar, 4 mm. (**F**) Beam profile measurement of skull effects. Color bar indicates normalized intensity values. Scale bars, 700 µm. (**G**) Simulated temperature map of a 460-kHz ultrasound beam continuously generated for 300 s. The color bar represents temperature, remaining relatively constant at 37 ºC. Scale bar, 8 mm. (**H**) Plot of temperature change measured across the central axial direction for 2, 25, 50, 100, 150, and 300 s. (**I**) Plot of temperature change at the surface of a CMUT device in operation.

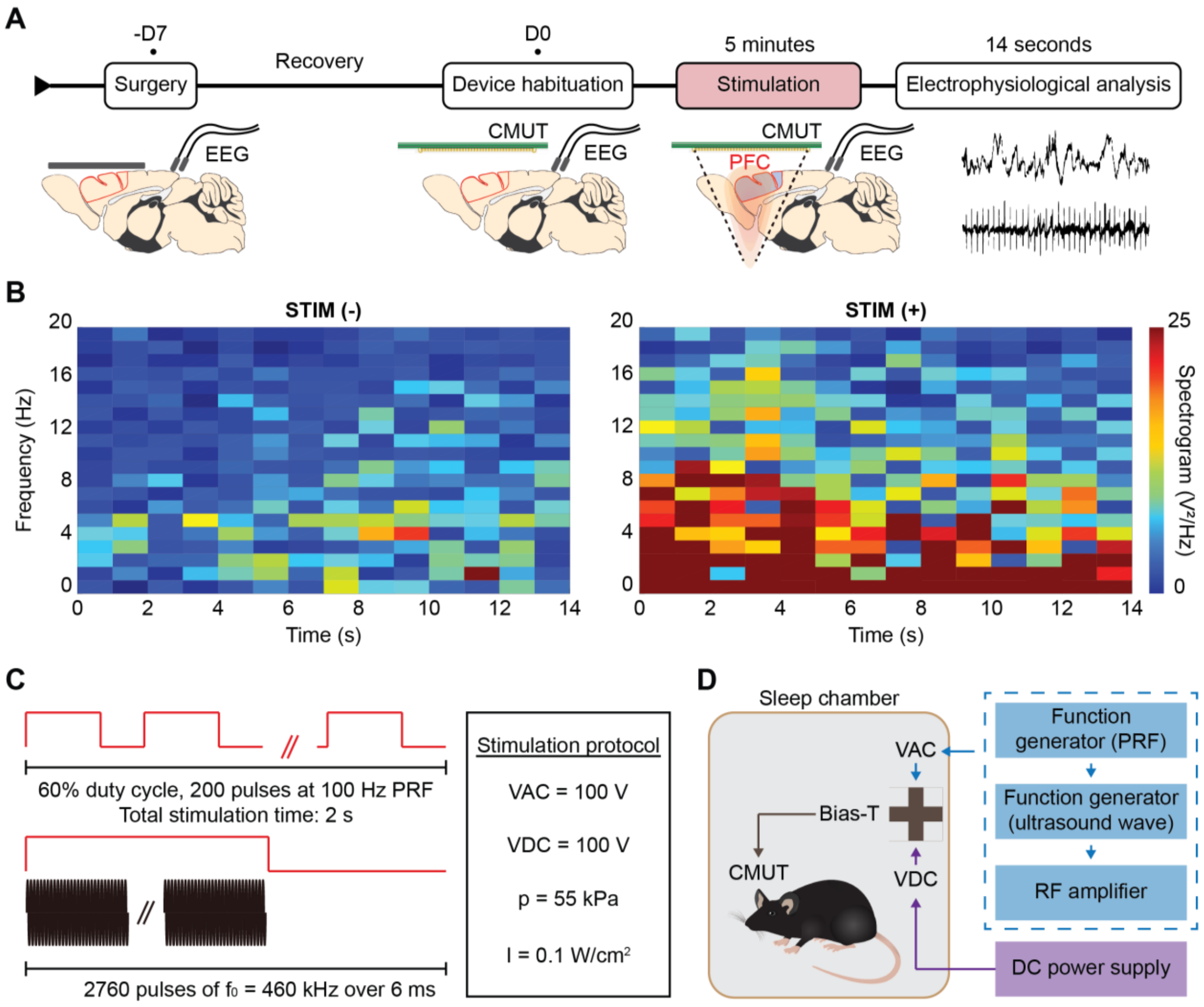

Figure S5. Pilot study on the effects of ultrasound stimulation. (**A**) Schematic of pilot study timeline with 5-min stimulation duration and 14-s EEG analysis. (**B**) Spectrogram results of pilot study demonstrating increased electroencephalography power in the 0.5 ~4 Hz band (NREM frequency) immediately following an acute 5-min ultrasound sonication session. Color bar indicates the power spectral density of the spectrogram. (**C**) Schematic of ultrasound waveform used for stimulation. (**D**) Schematic of *in vivo* ultrasound stimulation setup.

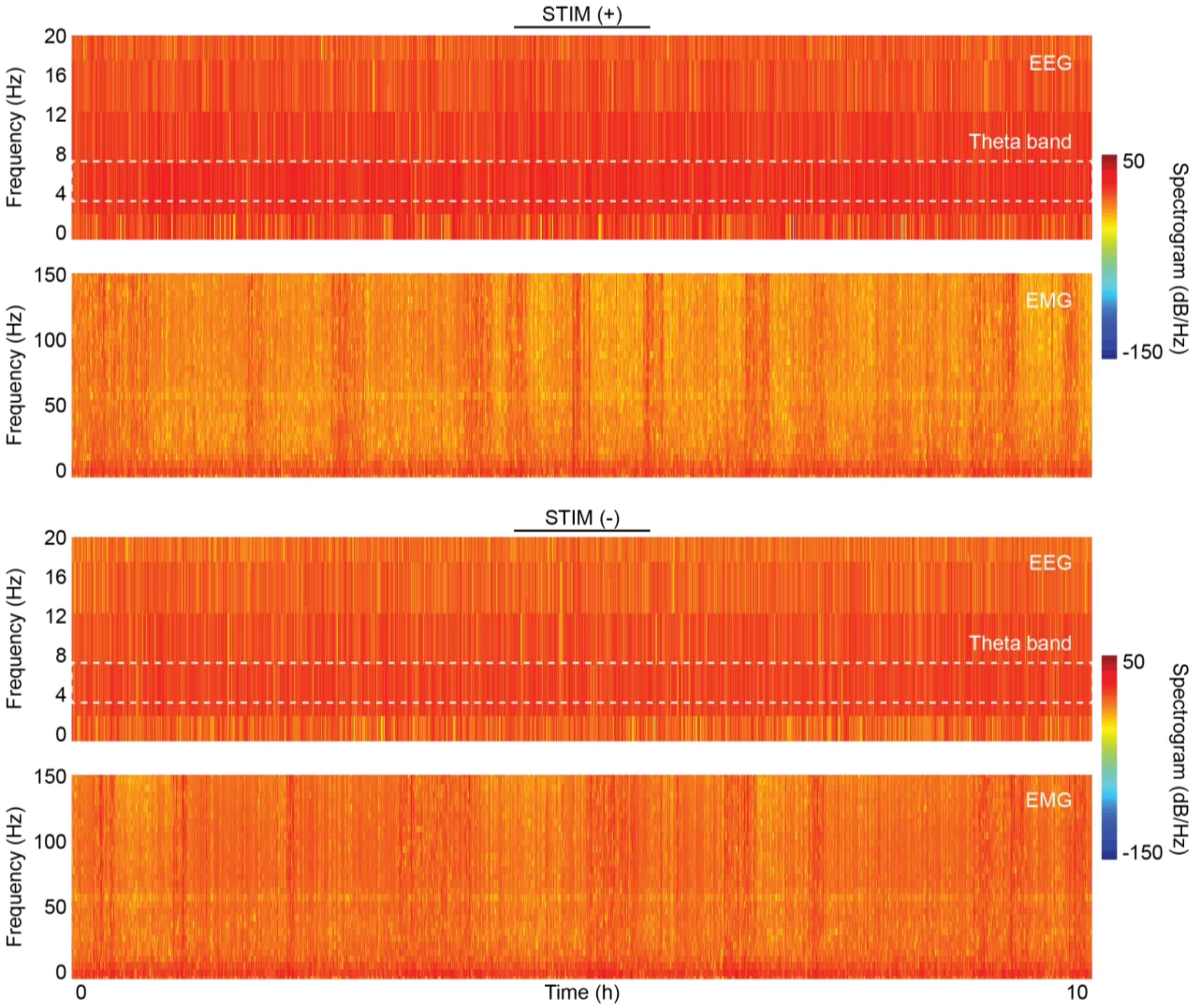

Figure S6. Spectrogram of STIM(+) and STIM(-) showing higher EEG power in the theta frequency band of STIM(+) mice.

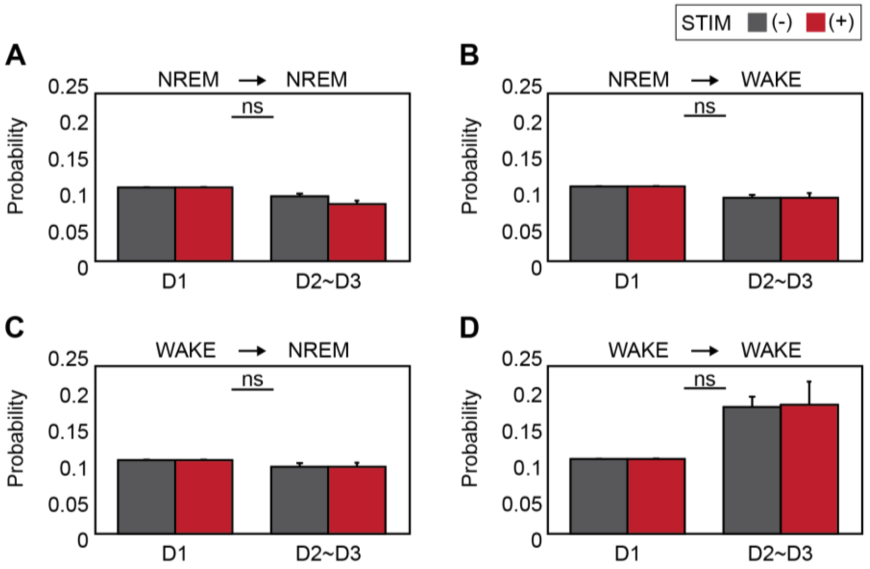

Figure S7. Probabilities of sleep state transition for NREM sleep and WAKE states. (**A**) to (**D**) Sleep state transition probabilities for NREM sleep maintenance (A), WAKE initiation from NREM (B), NREM initiation from WAKE (C), and WAKE state maintenance (D). ns, not statistically significant.

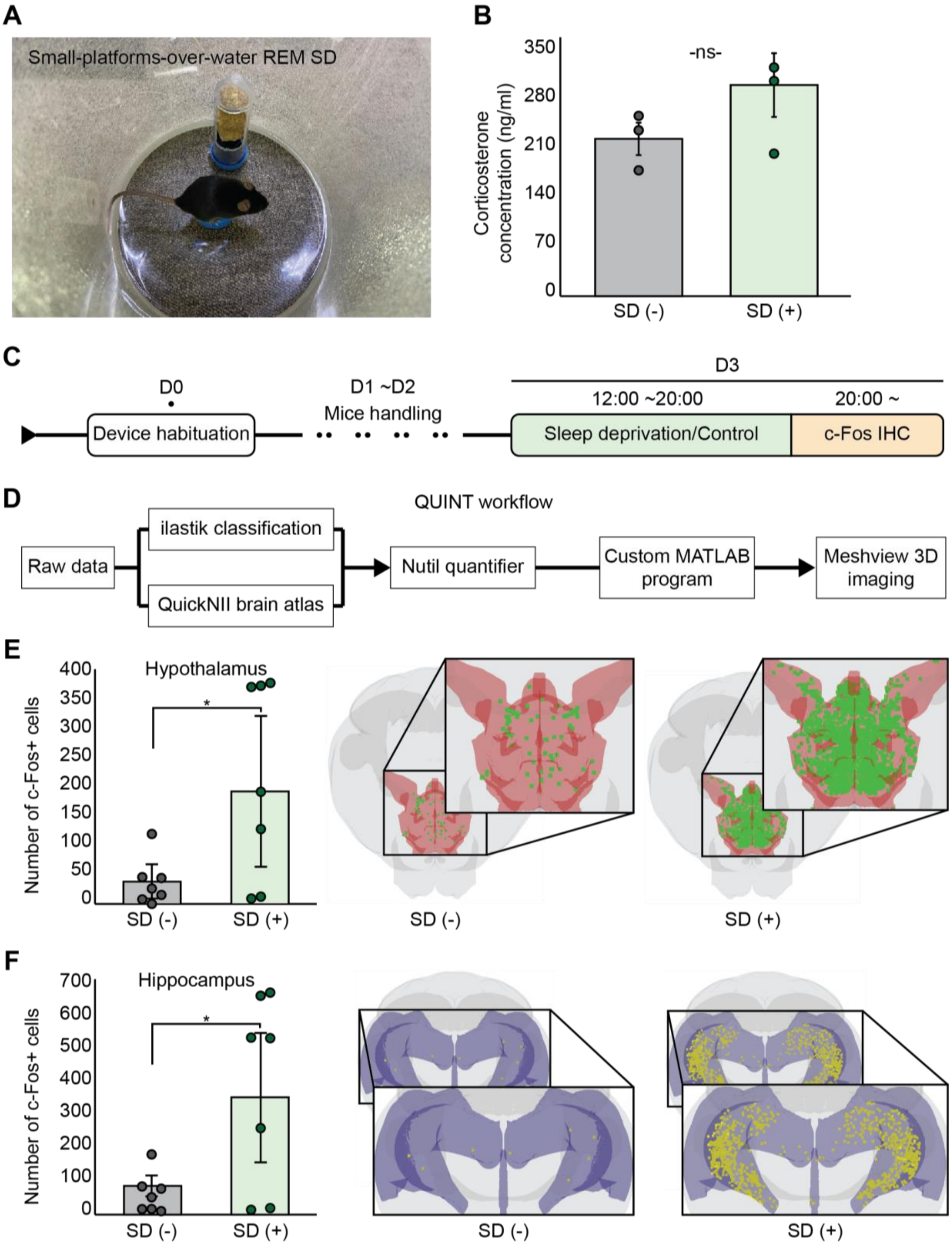

Figure S8. Verification of sleep deprivation protocol via circulating corticosterone level and c-Fos immunohistochemistry. (**A**) Small-platforms-over-water setup for REM sleep

deprivation. (**B**) Circulating corticosterone concentration for SD(-) and SD(+) mice. ns, not statistically significant. (**C**) Experimental timeline for c-Fos IHC in SD(-) and SD(+) mice. (**D**) Schematic of QUINT workflow for semi-automated c-Fos image analysis. (**E**) and (**F**) Number of c-Fos$^+$ cells in the hypothalamus (E) and hippocampus (F), and corresponding 3D reconstruction using Meshview. *P<0.05, two-sided unpaired Student's t-test, *n=14*.

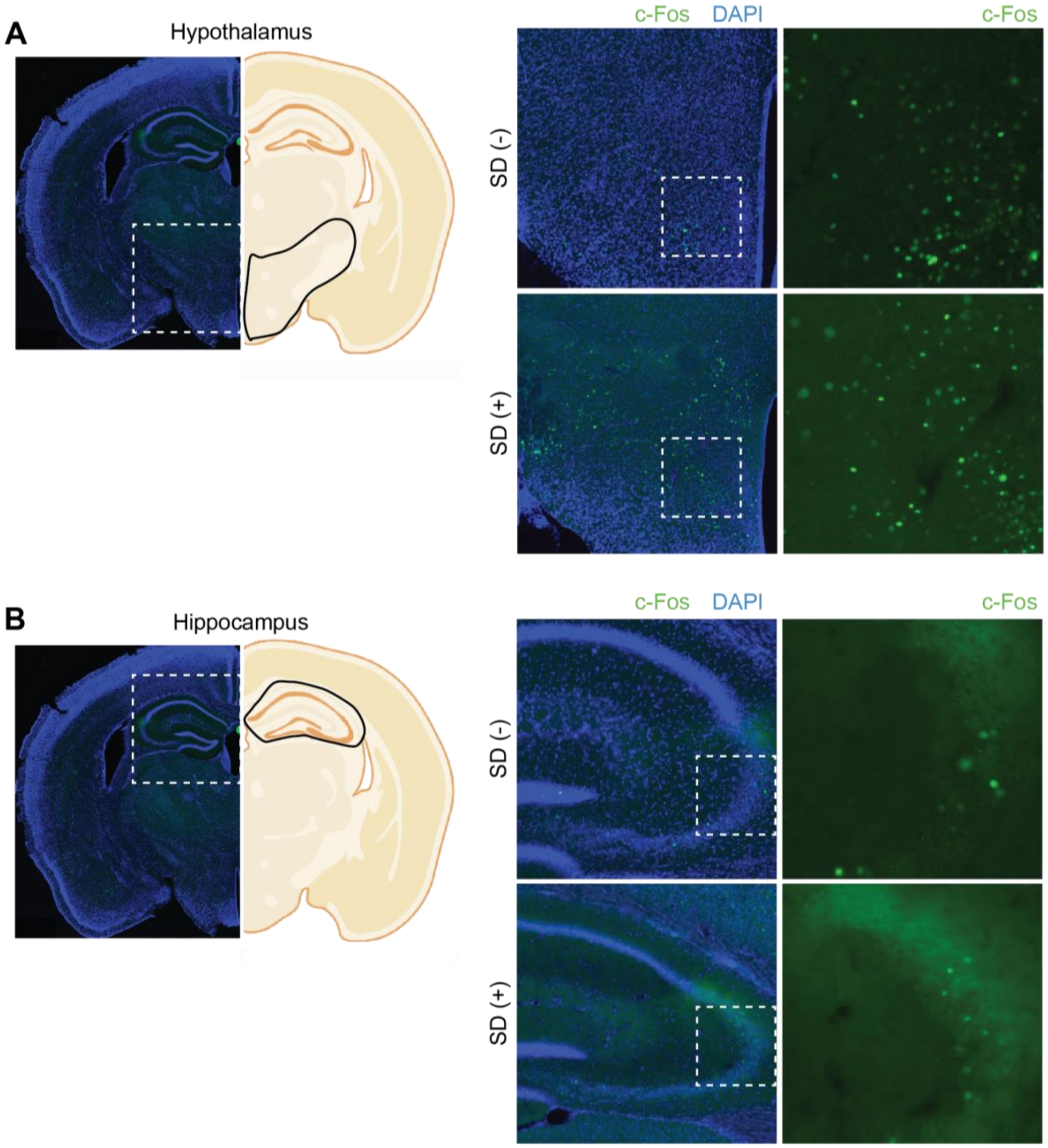

Figure S9. Microscopic images of brain slices for c-Fos immunohistochemistry. (**A**) and (**B**) Representative c-Fos and DAPI-stained brain slices of the hypothalamus (A) and hippocampus (B) for SD(-) and SD(+) mice.

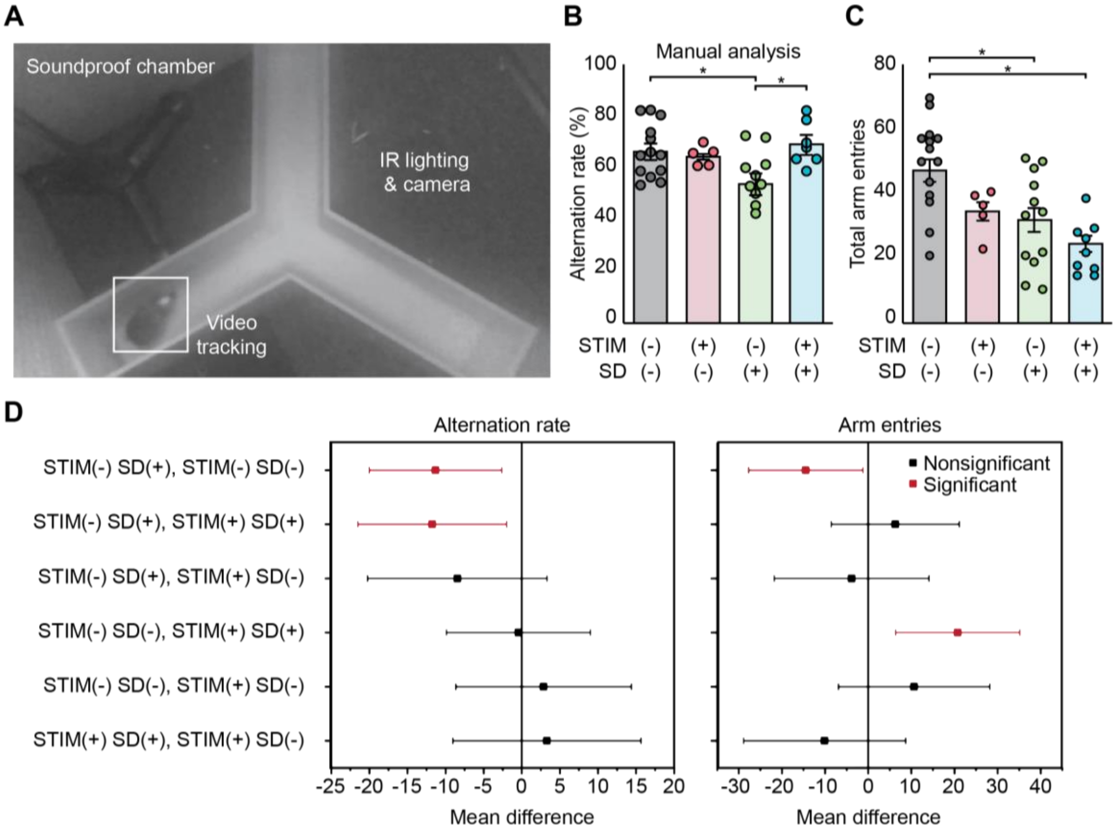

Figure S10. Y-maze setup and manual spontaneous alternation behavior analysis. (**A**) Infrared image of Y-maze setup inside a soundproof chamber with video recording. (**B**) Manual video analysis of spontaneous alternation rate in stimulated SD(-) and SD(+) mice. *P<0.05, one-way ANOVA with Tukey's post-hoc test, *n=40*. (**C**) Total number of arm entries for stimulated SD(-) and SD(+) mice. *P<0.05, one-way ANOVA with Tukey's post-hoc test, *n=40.* (**D**) Multiple comparison plots using experimental group as the factor for (left) alternation rate and (right) arm entries using one-way ANOVA with Tukey's post-hoc test.

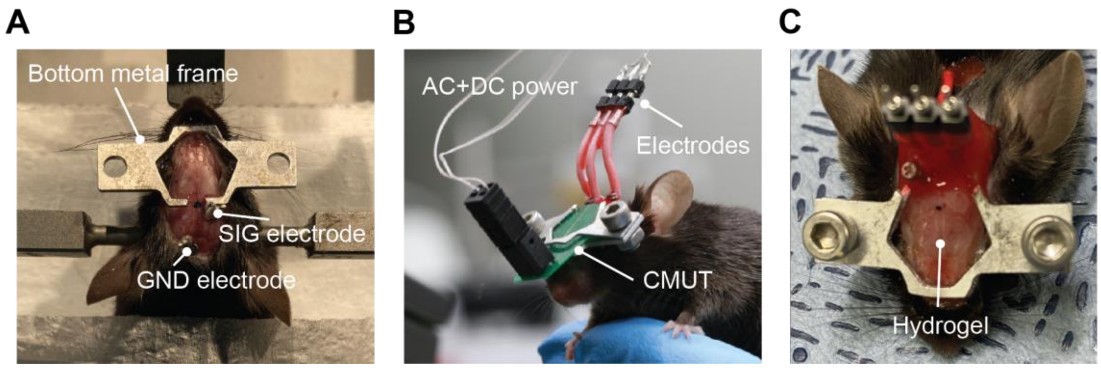

Figure S11. Mice surgical procedure and freely moving *in vivo* setup with CMUT device. (**A**) Surgical procedure of a bottom metal frame insert and EEG electrode implantation. (**B**) Freely moving mouse with CMUT device inserted between metal frames and surgically-implanted electrodes for electrophysiological monitoring. (**C**) Intact and functional hydrogel for ultrasound coupling after a 10-h *in vivo* experimental cycle.
